# Construction of DNA Tools for Hyperexpression in *Marchantia* Chloroplasts

**DOI:** 10.1021/acssynbio.0c00637

**Published:** 2021-06-07

**Authors:** Eftychios Frangedakis, Fernando Guzman-Chavez, Marius Rebmann, Kasey Markel, Ying Yu, Artemis Perraki, Sze Wai Tse, Yang Liu, Jenna Rever, Susanna Sauret-Gueto, Bernard Goffinet, Harald Schneider, Jim Haseloff

**Affiliations:** † Department of Plant Sciences, University of Cambridge, Downing Street, Cambridge CB2 3EA, U.K.; ‡ College of Life and Environmental Sciences, Hangzhou Normal University, Hangzhou 311121, China; § Fairy Lake Botanical Garden & Chinese Academy of Sciences, Shenzhen, Guangdong 518004, China; ∥ Department of Ecology and Evolutionary Biology, University of Connecticut, Storrs, Connecticut 06269-3043, United States; ⊥ Center for Integrative Conservation, Xishuangbanna Tropical Botanical Garden, Chinese Academy of Sciences, Menglun, Yunnan 666303, China

**Keywords:** *Marchantia*, plant, chloroplast, plastome, transcriptome, gene assembly

## Abstract

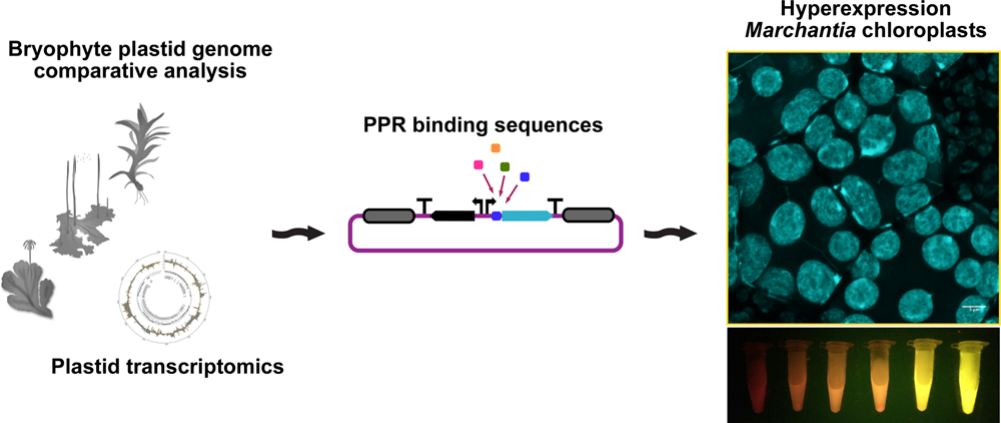

Chloroplasts are attractive platforms for synthetic biology applications since they are capable of driving very high levels of transgene expression, if mRNA production and stability are properly regulated. However, plastid transformation is a slow process and currently limited to a few plant species. The liverwort *Marchantia polymorpha* is a simple model plant that allows rapid transformation studies; however, its potential for protein hyperexpression has not been fully exploited. This is partially due to the fact that chloroplast post-transcriptional regulation is poorly characterized in this plant. We have mapped patterns of transcription in *Marchantia* chloroplasts. Furthermore, we have obtained and compared sequences from 51 bryophyte species and identified putative sites for pentatricopeptide repeat protein binding that are thought to play important roles in mRNA stabilization. Candidate binding sites were tested for their ability to confer high levels of reporter gene expression in *Marchantia* chloroplasts, and levels of protein production and effects on growth were measured in homoplastic transformed plants. We have produced novel DNA tools for protein hyperexpression in this facile plant system that is a test-bed for chloroplast engineering.

Chloroplasts are the semiautonomous organelles responsible for the capture of light energy through the conversion of CO_2_ to organic molecules in plants. The genomes of these plastids are small and highly conserved, present at a high copy number per cell, and not subject to gene silencing. Foreign proteins have been produced in chloroplasts at high levels, sometimes reaching a major proportion of the total soluble proteins in transformed plants.^1−3^ However, previous attempts to harness this capacity for routine hyperexpression (>1% soluble protein) have been irregular and sporadic. The primary reasons for this lack of application are the relatively small number of species with established methods for chloroplast transformation, the slow pace and inefficiency of plastid transformation, and the inconsistent levels of gene expression between experiments.

*Marchanti*a *polymorpha* is one of the few land plant species for which chloroplast transformation is well established.^4,5^*Marchantia* has a series of characteristics that make it an ideal platform for chloroplast engineering.^6^ The dominant phase of the life cycle is haploid, it has simple requirements for culture (*i.e.*, no need for glasshouses and expensive or specialized media or infrastructure for plant growth), offers the benefits of spontaneous regeneration at high efficiency in the absence of phytohormones, fast selection for homoplasmy (transplastomic plants can be isolated within 8 weeks^7^), and simple microscopic observation. We have developed an open-source DNA toolkit, called OpenPlant kit, for facile engineering of the plastid genome in *Marchantia*(7) (Figure 1). The toolkit is based on Loop assembly,^8^ a Type IIS method for DNA construct generation that employs a recursive strategy to greatly simplify the process of plasmid assembly. It allows rapid and efficient production of large DNA constructs from DNA parts that follow a common assembly syntax. Unlike other systems that require elaborate sets of vectors, Loop assembly requires only two sets of four complementary vectors. In a series of reactions, standardized DNA parts can be assembled into multitranscriptional units. *Marchantia* shows great promise as a simple and facile test-bed for chloroplast engineering, but little is known of the cis-regulatory elements required to fully exploit the capacity of plastids for high and sustained levels of gene expression.

**Figure 1:**
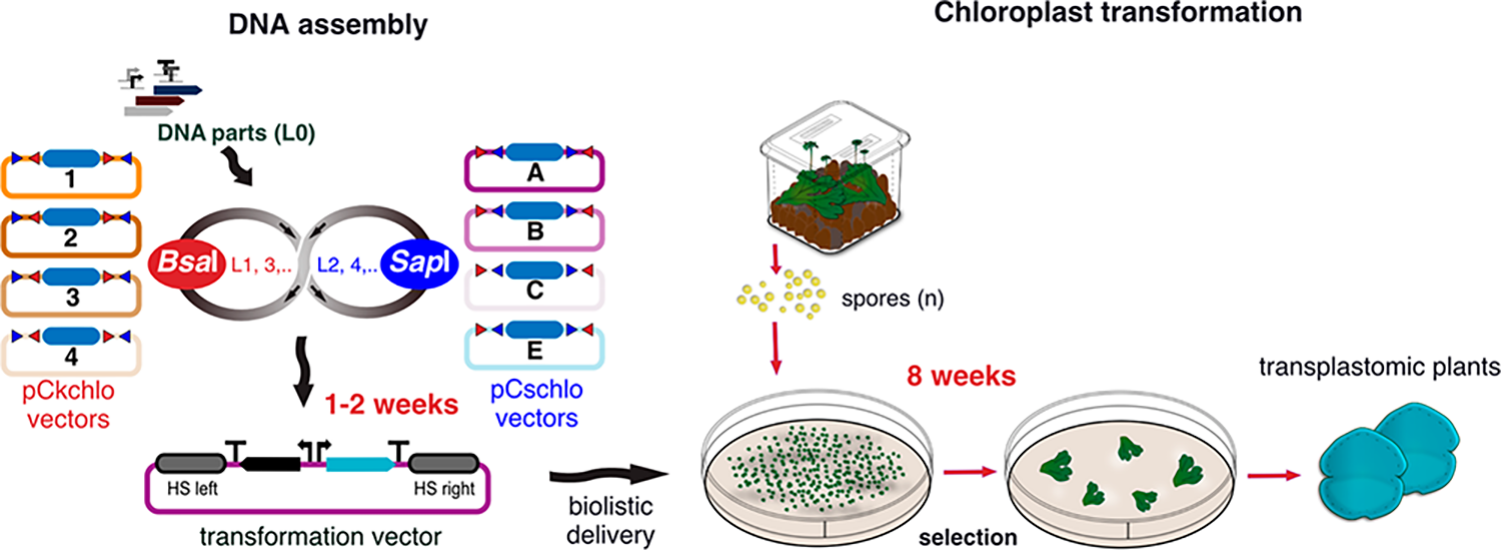
*Marchantia* chloroplast Loop assembly and transformation overview. Level 0 (L0) DNA parts are assembled in Level 1 (L1) transcription units (TUs) into one of the four pCkchlo vectors, depicted with numbered circles, by *Bsa*I-mediated Type IIS assembly (sequential restriction enzyme digestion and ligation reactions). L1 TUs are assembled to Level 2 (L2) multi-TUs into one of the four pCschlo vectors by *Sap*I-mediated Type IIS assembly. The recursive nature of Loop assembly means that this workflow can be repeated for higher level assemblies (L3, L4, *etc*.). L2 constructs can be generated from L0 parts in one–two weeks. *Bsa*I and *Sap*I recognition sites are represented as red and blue triangles, respectively. HS: homologous sequences, bent arrows: promoters, arrows: coding sequences and “T”: terminators. Blue filled rectangle: LacZ bacteria selection cassette. Microboxes are used to produce spores (n: haploid). Seven day old sporelings are bombarded with DNAdel nanoparticles coated with the desired DNA construct. After bombardment, sporelings are plated on selective media, and after four weeks successful transformants start to be visible. After a second round of selection (four weeks), gemmae are produced and can be tested for homoplasmy by genotyping PCR.

Past attempts to build more efficient vectors for chloroplast gene expression have focused on increasing the efficiency of transcription, translation initiation, and codon usage. However, recent work has led to a breakthrough in understanding the important roles of post-transcriptional processing and mRNA stability in conferring high levels of gene expression in chloroplasts.^9,10^ Plastid RNA transcripts are subject to a series of complex processing steps that are primarily mediated by nucleus-encoded factors, including pentatricopeptide repeat (PPR) containing proteins. The PPR proteins are a large family of RNA-binding proteins that have undergone a substantial expansion in plants^11^ and are required for stabilization of mRNAs by protection from exonuclease activity in the plastid.^9,12^ The sequence-specific RNA-binding properties and defined target sites for these proteins make them excellent candidates as artificial regulators of RNA degradation, in addition to being used as highly effective tools for enhancing gene expression in chloroplasts.^9,10^ Post-transcriptional regulation of chloroplast mRNAs in *Marchantia* is relatively simple compared to vascular plants. For example, the *Marchantia* nuclear genome encodes 75 PPR proteins^13^ directed to chloroplasts and mitochondria, while the *Arabidopsis* and rice genomes encode over 450 and 600 PPR proteins, respectively.^14^ Additionally, no evidence of PPR protein-mediated base editing has been found in *Marchantia* chloroplast transcripts.^15^

In order to identify conserved PPR-binding sequences in the 5’ untranslated regions (5’UTRs) of *Marchantia* chloroplast genes we conducted a transcriptional analysis of the *Marchantia* chloroplast, and also examined an expanded range of bryophyte plastid genomes. This study provides the first description of chloroplast transcription patterns in a liverwort, and comparisons within this under-studied group of land plants. It has also produced a variety of new DNA tools that enable the generation of plants capable of hyperexpression of proteins in the *Marchantia* facile model system.

## Results and Discussion

### *Marchantia* Chloroplast Transcriptome Analysis

We previously generated a high-quality plastid genome assembly for the *M. polymorpha* Cam1/2 isolates using next generation sequencing data (Genbank accession: MH635409)^7^ (Figure S1a). We conducted this assembly to resolve a taxonomic misidentification of the source of the reference plastid genome (Genbank accession NC_001319.1), which likely originated from the related species *Marchantia paleacea*.^16^ The plastid genome of *M. polymorpha* Cam1/2 is 120,314 bp and contains 123 annotated genes,^17^ which are mainly involved in photosynthesis, electron transport, transcription, and translation. A small number of genes with more specific functions are also present, such as the *chlL* gene involved in chlorophyll biosynthesis.^17^ Comparison of the *Marchantia* plastid genome with those of angiosperms, such as *Arabidopsis* and tobacco, reveals remarkable conservation both in regards to gene number, functions and local organization^18−20^ (Figure S1b).

**Figure 2:**
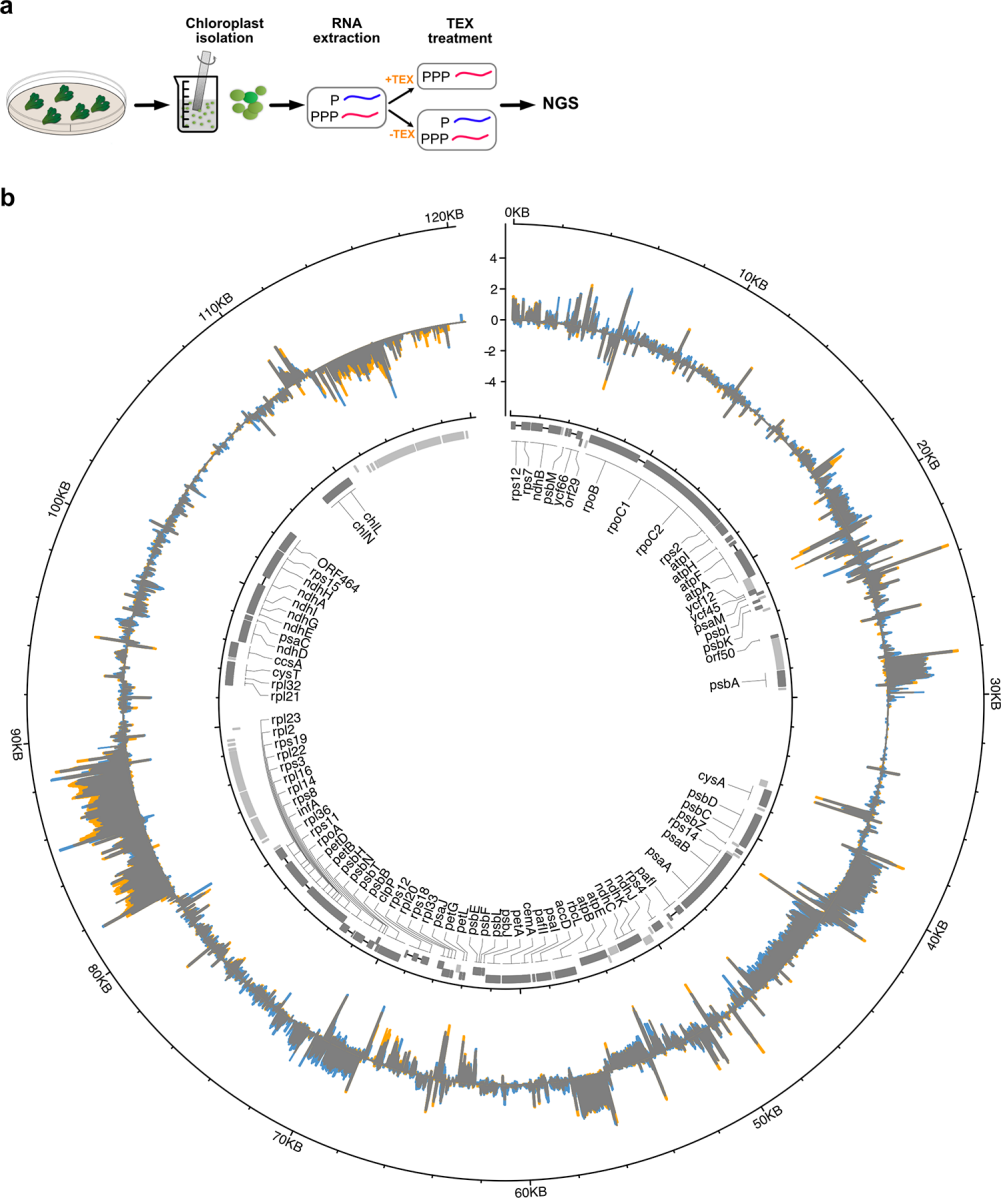
(a) Outline of dRNaseq pipeline. Plant tissue was collected and homogenized. Intact chloroplasts were isolated from homogenized plant tissue, RNA was extracted and then subjected to treatment with the Terminator 5′ phosphate dependent exonuclease (TEX) enzyme. TEX degrades RNAs with a 5′ monophosphate (processed transcripts) but not those with a 5′ triphosphate (primary transcripts). Consequently, comparison of next generation sequencing libraries generated from TEX treated (TEX+) and nontreated (TEX−) samples can be used to identify the protected primary transcripts and their TSSs. The identification of TSS allows more accurate mapping of promoter regions. (b) dRNaseq in *Marchantia*. Median circle: Reads of samples with TEX treatment (TEX+ libraries) and without TEX treatment (TEX– libraries), mapped on *M. polymorpha* Cam1/2 accession plastid genome (MH635409). Forward strand coverage faces outward, reverse strand coverage faces inward. *Y*-axis: log10 coverage per million mapped reads. Blue: excess TEX– coverage (TEX– enrichment), Orange: excess TEX+ coverage (TEX+ enrichment), Gray: TEX– = TEX+. Inner circle depicts the gene organization of the *Marchantia* plastid genome. Protein coding genes are shown in dark gray boxes; boxes show coding sequences and lines introns. Noncoding genes are shown as light gray boxes. Boxes for genes encoded clockwise face outward, those encoded counter clockwise strand genes face inward. Gene names are shown for protein coding genes in the center.

Recent experiments have demonstrated the crucial importance of both promoter identity and adjacent 5′UTRs for initiating and stabilizing high levels of transcription in chloroplasts.^10,21^ In order to better understand which sequences might be useful for engineering high levels of gene expression, we employed differential RNA sequencing (dRNaseq),^22^ which allowed identification of primary transcripts in extracted chloroplast RNAs. This technique was initially developed for prokaryotic organisms but has also successfully been applied to barley chloroplasts.^22,23^ RNAs isolated from *Marchantia* chloroplasts were treated with Terminator 5′ phosphate dependent exonuclease (TEX) in order to selectively degrade RNAs with 5′ monophosphate termini, while primary transcripts with 5′ triphosphate termini are resistant to degradation (Figure 2a). Treated and untreated RNA populations were sequenced to locate transcription start sites (TSS), and putative promoter and 5′UTRs. The main goals of these experiments were (i) to identify highly transcribed regions of the *Marchantia* plastid genome, (ii) to locate transcription start sites of mRNAs that accumulate to high levels, and (iii) to screen for conserved sequences that might indicate important features that could be incorporated into synthetic promoter and mRNA elements to promote high levels of protein expression.

Short sequence reads (75 bp) were obtained from TEX treated and untreated RNA samples and mapped onto the plastid genome of *M. polymorpha* accession Cam1/2 (MH635409) (Figure 2b and Figure S2 and Table S1). The levels of transcript abundance could be observed. These were mapped onto different regions of the plastid genome, with evident polarity that reflected the directions of transcription across transcribed genes and operons.

**Figure 3:**
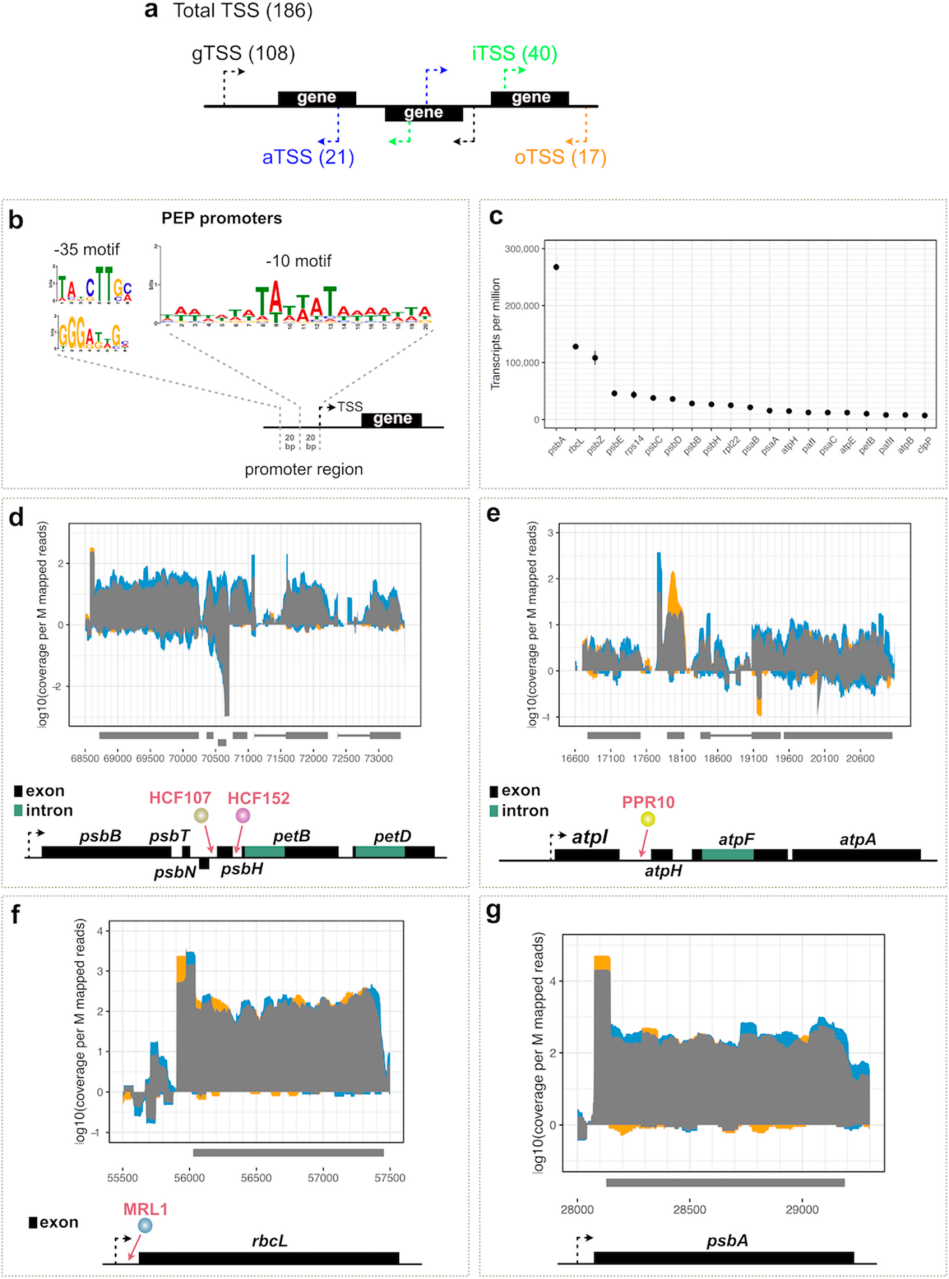
(a) Graphical summary of different species of TSSs identified in the *Marchantia* plastid genome using dRNaseq. A total of 186 potential TSSs were identified, with the most abundant species associated with tRNAs. The identified TSSs could be further grouped into four categories based on their genomic location: (i) gene TSSs (gTSSs) found within a region upstream of annotated genes, (ii) internal TSSs (iTSSs) found within annotated genes and giving rise to sense transcripts, (iii) antisense TSSs (aTSSs) located on the opposite strand within annotated genes and giving rise to antisense transcripts, and (iv) orphan TSSs (oTSSs). In total 108 gTSSs, 40 iTSSs, 21 aTSSs, and 17 oTSSs were mapped. (b) MEME^31^ analysis discovered a −10 PEP consensus element upstream of 140 TSSs (e-value 5.3 × 10^–028^). Two −35 PEP consensus motifs were predicted in 25 out of the 140 sequences. Top: 16 sequences (e-value: 2.5 × 10^+001^). Bottom: Nine sequences (e-value: 8.4 × 10^–002^). (c) Top 20 genes, excluding tRNAs and rRNAs, with the highest expression levels (TPM) in *Marchantia* chloroplast. (d–g) Primary transcript enriched (TEX+ libraries) and nonenriched (TEX– libraries) mapped on the genomic location of (d) Mp-*psbB* operon and (e) large Mp-*atp* operon, (f) Mp-*rbcL*, and (g) Mp-*psbA*. *X*-axis: genomic position. *Y*-axis: coverage per million of mapped reads. Blue: excess TEX– coverage (TEX– enrichment). Orange: excess TEX+ coverage (TEX+ enrichment). Gray: TEX– = TEX+. Operon maps are depicted below the graphs. (d) The *psbB* operon comprises five genes: *psbB*, *psbT*, *psbH*, *petB*, and *petD*. Each of the *petB* and *petD* genes contains an intron. The *psbN* gene, which is encoded in the intercistronic region between *psbH* and *psbT*, is transcribed in the opposite direction. In *Marchantia* we identified a TSS 144 bp upstream the *psbB* gene, 47 bp upstream the *psbN* gene, 36 bp upstream the *psbH* gene, and 43 bp upstream the *petB* gene. In *Arabidopsis* the HCF152 PPR protein binds to a sequence located in the 5′UTR of the *petB* chloroplast gene stabilizing RNA transcripts against 5′ → 3′ exonuclease degradation.^32^ The HCF107 protein binds upstream *psbH* to stabilize the *psbH* transcript and activates *psbH* translation.^42^ (e) The large *atp* operon is composed of four genes: *atpI*, *atpH*, *atpF*, and *atpA*. In *Marchantia* we identified a TSS 73 bp upstream the *atpI* gene and an internal TSS 141 bp upstream the *atpH* gene. In maize, the PPR10 protein binds to a sequence located in the 5′UTR of the *atpH* chloroplast gene and has been found to play a role in controlling translation by defining and stabilizing the termini, protecting them from exonucleases.^44^ (f) We identified a TSS 124 bp upstream the *rbcL* gene. In *Arabidopsis* the MRL1 PPR protein binds to a sequence located in the 5′UTR of the *rbcL* chloroplast gene, acting as a barrier to 5′ → 3′ degradation.^43,44^ (g) We identified a TSS 54 bp upstream the *psbA* gene.

We manually assigned a total of 186 potential TSSs to locations on the *Marchantia* chloroplast genome (Figure 3a and Table S2). The identified TSSs could be grouped into four categories based on their genomic location: (i) gene TSSs (gTSSs) found within a region upstream of annotated genes, (ii) internal TSSs (iTSSs) found within annotated genes and giving rise to sense transcripts, (iii) antisense TSSs (aTSSs) located on the opposite strand within annotated genes and giving rise to antisense transcripts, which could indicate the synthesis of noncoding RNAs; and (iv) orphan TSSs (oTSSs). In total, we mapped 108 gTSSs, 40 iTSSs, 21 aTSSs, and 17 oTSSs (Figure 3a).

The most abundant gTSSs corresponded to tRNA genes. The *Marchantia* plastid genome encodes 31 unique tRNAs (tRNA),^17^ five of which are present in two copies in the inverted repeat (IR) regions. Given that the genome contains only 123 genes,^17^ the number of identified TSSs exceeded expectations, especially considering that some are likely encoded in cotranscribed operons. The experimental approach can be confounded by post-transcription processing or degradation, or low abundance of primary transcripts.

### Characterization of Active Promoters and Transcripts

Plastid transcription is mediated by two distinct RNA polymerases: the eukaryotic nuclear encoded RNA polymerase (NEP) and the prokaryote-like plastid encoded RNA polymerase (PEP), which is retained from the cyanobacterial endosymbiont.^24^ PEP recognizes bacterial type promoters that contain conserved domains at positions −35 and −10 (TATA),^25^ whereas NEP recognizes promoters that have a core sequence “YRTA” (where Y is cytosine or thymine, and R is guanine or adenine) motif in close proximity to the transcription start site.^25,26^ However, many genes can be transcribed by both. In general, PEP promoters appear to be much stronger than NEP promoters, and highly expressed genes in the plastid genome (*e.g.*, most photosynthesis genes) are usually transcribed from PEP promoters.^25^ For this reason, PEP promoters have been predominantly used to drive the expression of plastid transgenes.

A limited number of promoters have been employed for transgene expression in chloroplasts, and mainly in systems such as tobacco and *Chlamydomonas*.^27,28^ These promoters are derived from highly expressed plastid genes, such as the large subunit of ribulose-1,5-bisphosphate carboxylase/oxygenase (RuBiSco) (*rbcL*), the photosystem II protein D1 (*psbA*) gene and the plastid rRNA operon, *rrn*. Only two studies have focused on promoter regions of plastid genes in *Marchantia*:^29^ analyzed the promoter region of the *psbD* gene and^30^ predicted the promoter regions of *psaA*, *psbA*, *psbB*, *psbE*, and *rbcL* genes based on sequence comparison of several plant species.

Studies in *Marchantia* have employed heterologous tobacco *psbA* and *prrn* promoters to drive expression of transgenes.^4,5^ The identification of *Marchantia* plastid gene TSSs has allowed precise characterization of the initiation sites for transcription, and the mapping of the 5′ termini of transcripts in a wide range of genes. These newly identified elements crucially expand the repertoire of available promoter parts to be considered when designing transgenes for *Marchantia* chloroplast engineering.

The 50-nucleotide regions upstream of the identified TSSs were screened for potential promoter motifs using the Multiple Expectation maximization for Motif Elicitation (MEME) tool.^31^ We found a −10 TAttaT motif located three to nine nucleotides upstream of the transcription start point for 140 predicted TSSs, similar to that found in barley^23^ (Table S3). Examination of the −35 region showed a lower degree of sequence conservation than the −10 box. Two −35 motifs were mapped in only 25 out of those 140 TSSs (Figure 3b).

To distinguish candidate DNA parts for high level gene expression, we used data from untreated dRNaseq samples and identified the 20 protein-encoding genes with the highest RNA accumulation in the *Marchantia* chloroplast. (Figure 3c). As was found in other plants,^28^ the *psbA* and *rbcL* genes have the highest mRNA transcript levels in *Marchantia* chloroplasts. The dRNaseq profiles of the promoter regions of these genes were examined in more detail. The genetic maps and transcript profiles of these regions are shown in Figure 3f, g. After TEX treatment, we observed an approximately 5-fold enrichment of reads mapped at the 5′ end of the primary transcript for *rbcL* and approximately 2.5-fold enrichment for *psbA*. The identified TSSs were located 124 bp and 54 bp upstream of the predicted start codons for *rbcL* and *psbA*, respectively.

### Operons

Many chloroplast genes, often functionally related, are organized in cotranscribed operons. Examples include the *psbB* operon and the two ATP synthase (*atp*) operons (the large *atpI*/*H*/*F*/*A* and the small *atpB*/*E* operon). Operons are usually transcribed as a unit and the transcripts processed to yield smaller monocistronic mRNAs. Operon processing is mediated by various factors that recognize particular operon noncoding sequences. These sequences harbor gene expression elements, such as PPR binding motifs, that are potentially useful for plastid engineering applications. As for promoters, the available information about operon structure and regulation in *Marchantia* is very limited.

The *psbB* operon comprises five genes encoding the photosystem II subunits CP47 (*psbB)*, T (*psbT*), and H (*psbH*) as well as cytochrome b6 (*petB*) and subunit IV (*petD*) of the cytochrome b6f complex. In *Arabidopsis* it is initially transcribed as a large precursor mRNA, which is extensively processed.^32^ Each of the *petB* and *petD* genes contains an intron, which is spliced during post-transcriptional modification. The *psbB* operon is regulated by more than one promoter (Figure 3d). In particular, the small subunit of photosystem II (*psbN*), which is encoded in the intercistronic region between *psbH* and *psbT*, is transcribed in the opposite direction by an additional promoter. In *Marchantia* we identified a TSS 144 bp upstream of the *psbB* gene, 47 bp upstream of the *psbN* gene, 36 bp upstream the *psbH* gene, and 43 bp upstream the *petB* gene.

The large *atp* operon is composed of four genes: *atpI*, *atpH*, *atpF*, and *atpA*. Plastid operons often have multiple promoters that enable a subset of genes to be transcribed within the operon.^33^ For example, this operon is transcribed by two PEP promoters in *Arabidopsis*, one upstream and one within the operon, and harbors four potential sites for RNA-binding proteins.^34^ In *Marchantia* we identified a TSS 73 bp upstream of the *atpI* gene and an internal TSS 141 bp upstream of the *atpH* gene (Figure 3e).

### Comparisons with Other Bryophyte Plastid Genomes

Over 4500 plastid genomes have been sequenced to date, and the overwhelming majority of these belong to angiosperm plants.^14,35,36^ Sequence comparisons between the plastid genomes of land plants have revealed gross gene rearrangements, but individual coding regions and a number of gene clusters are recognizably conserved. In addition, certain cis-regulatory sequences, such as PPR-binding sites, are conserved and often located near the 5′ termini of mRNA transcripts.^37^ However, the small size and apparent sequence redundancy of the sequences makes them difficult to identify by comparison between divergent species. At the initial phase of our investigation, only eight bryophyte plastid genomes were publicly available. To overcome this limitation, we expanded the sampling to 51 plastid genomes from bryophytes, and used comparative genomics to screen the *Marchantia* plastid genome for potential regulatory sequences.

**Figure 4:**
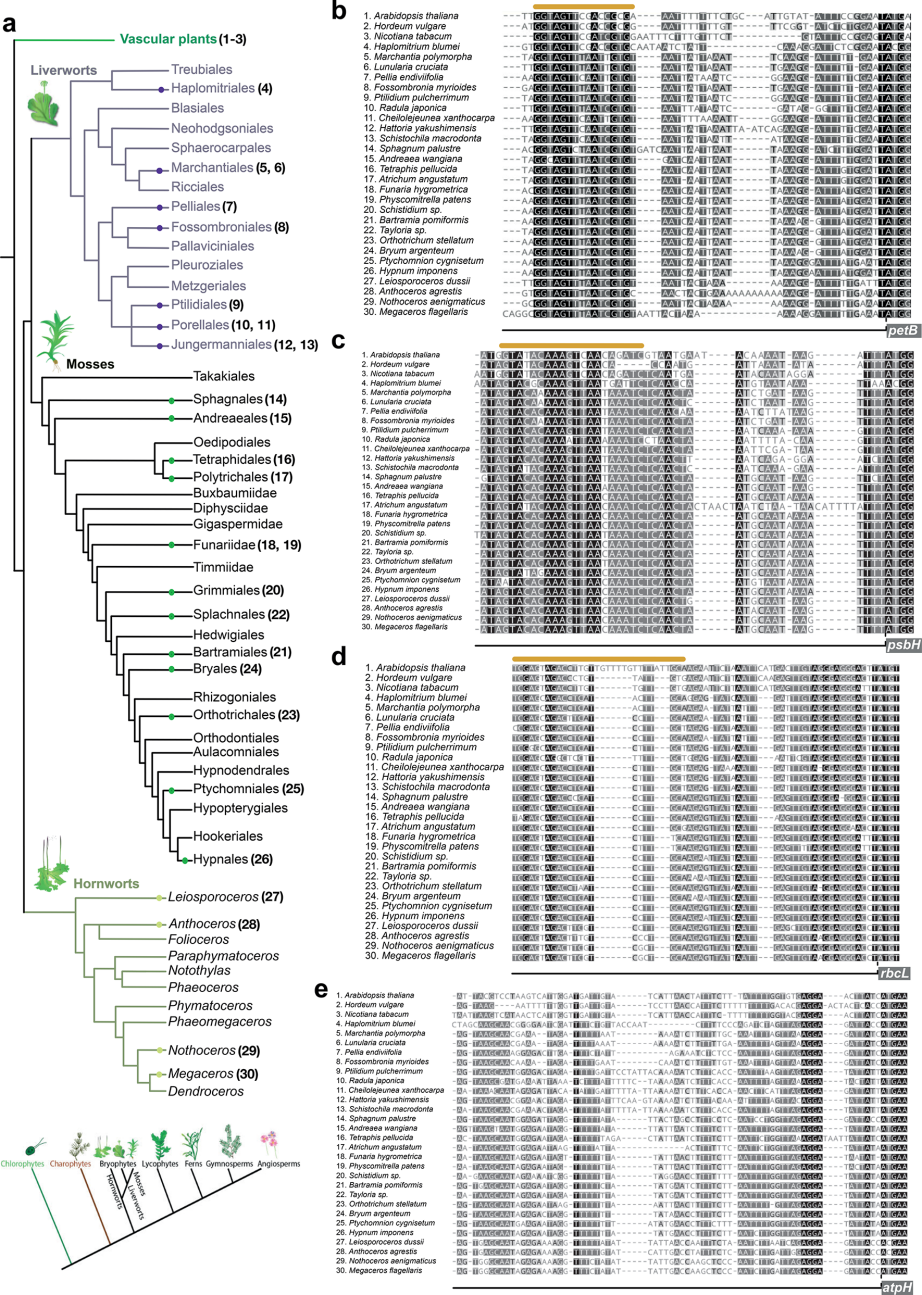
(a) Bryophyte phylogeny modified from ref (40) using the most recent phylogenetic inference about the relationship of bryophytes.^38^ Numbers next to Order names indicate sampled species, which were included in our analysis. Bottom: Land plant phylogenetic tree based on ref (38) with bryophytes being monophyletic and hornworts being sister to mosses and liverworts. (b–e) Multiple sequence alignments, using MUSCLE,^60^ of upstream nucleotide sequences of *petB* (b), *psbH* (c), *rbcL* (d), and *atpH* (e) genes from 27 different bryophyte species and three angiosperms. Numbers next to species names correspond to the phylogenetic Order in (a). ATG site is indicated with a dashed line. Coding sequence is indicated with a gray box. The predicted PPR binding site is highlighted by an orange line above. The coloring used for that column depends on the fraction of the column that is made of letters from this group. Black: 100% similar, dark-gray: 80–100% similar, lighter gray: 60–80% similar, white: less than 60% similar.

We determined the complete sequences of 26 liverwort plastid genomes, 16 moss genomes and one hornwort genome. We also included in our analysis two recently published hornwort plastid genomes^38^ and six published bryophyte plastid genome sequences (Table S4 and S5), as well as three angiosperm plastid sequences for reference. The data set comprised representatives of all three classes of liverworts, namely Haplomitriopsida, Marchantiopsida, and Jungermanniopsida.^39^ In summary, we included representatives of seven of the 15 liverwort orders, 12 of the 29 moss orders^40^ and three of the five hornwort orders^41^ currently recognized (Figure 4a and Table 1). Comparison of the newly generated bryophyte plastid genomes further supports the observation of a remarkable conservation of plastid genome structure among land plants.^40^

**Table 1:** Sampling of Land Plant Plastid Genomes Employed in This Study

lineages	orders	families	orders sampled	families sampled
Hornworts	5	11	3	3
Liverworts	15	87	7	21
Mosses	29	109	12	13
Angiosperms	64	418	3	4

### Identification of Putative PPR Protein Binding Sites

In order to identify conserved sequences that could be important for mRNA function in the chloroplast, we performed a phylogenetic comparison of mRNA sequences (up to ∼100 bp) upstream of the predicted initiator codon of the highly expressed *petB*, *psbH*, *rbcL* and *atpH* coding regions (Figure 4 b–e). It is known that similar regions within the corresponding angiosperm mRNA sequences encode binding sites for specific PPR proteins. The HCF152 PPR protein binds to a sequence located in the 5′UTR of the *petB* chloroplast mRNA. It has been experimentally demonstrated that binding of the protein to RNA transcripts stabilizes them against 5′ → 3′ riboexonuclease degradation in *Arabidopsis*.^32^ We also included in our analysis the High Chlorophyll Fluorescence 107 (HCF107) protein, which is a member of the family of PPR proteins that contain domains similar to histone acetyltransferases (HAT). HCF107 stabilizes the *psbH* transcript and activates *psbH* translation.^42^ The MRL1 PPR protein binds to a sequence located in the 5′UTR of the *rbcL* chloroplast gene. In *Arabidopsis*, MRL1 is necessary for the stabilization of the *rbcL* processed transcript, likely because it acts as a barrier to 5′ → 3′ degradation.^43^ The PPR10 protein binds to a sequence located in the 5′UTR of the *atpH* chloroplast gene and has been found to play a role in controlling translation by defining and stabilizing the 5′ terminus, protecting it from exonuclease activity.^44^ The *Marchantia* nuclear genome encodes 75 PPR proteins.^13,44^ We used Orthofinder^45^ to identify homologues of High Chlorophyll Fluorescence 152 (HCF152), Maturation of *rbcL* 1 (MRL1), PPR10 and HCF107 (Figure S3). To further confirm functional conservation of the identified homologues in *Marchantia* we compared the fifth and last amino acids of each PPR motif in *Arabidopsis* or maize and *Marchantia* (Figure S3). This comparison allows the prediction of the PPR binding site sequence. *Marchantia* HCF152 and two MRL1 putative homologues seem to bind similar sequences with those of *Arabidopsis*. However, for the *Marchantia* PPR10 putative homologue, the predicted binding sequence differs from that of maize indicating functional divergence.

We used the new bryophyte plastid genome alignments to search for conserved mRNA sequence motifs across both bryophyte and angiosperm plant species. Figure 4b–e shows the alignments of 30 plastid genome segments from bryophytes and key angiosperm species (alignments for all bryophyte species used in this study in Figure S4). The alignments correspond to the 5′UTR sequences of *petB*, *psbH*, *rbcL* and *atpH* mRNAs. The relevant PPR protein binding sites have been experimentally determined in certain angiosperms, and the binding footprints are indicated.^37^ These footprints coincide with conserved nucleotide sequences at the binding site. These sequences appear highly conserved across the angiosperms and bryophytes for the *Marchantia petB*, *psbH*, and *rbcL* chloroplast mRNAs, although not for *atpH* mRNA.

The nucleotide sequence similarity of these putative binding sites, and existence of homologous PPR proteins in *Marchantia* suggests that the functional relationship between nuclear-encoded PPR proteins and regulation of chloroplast mRNA stability may be conserved for (at least) *petB*, *psbH*, and *rbcL* across the land plants. Further, these putative PPR protein binding sites in *Marchantia* might be transplanted into engineered chloroplast genes and confer improved mRNA stability. We built and tested hybrid genes to test this hypothesis.

### Creating Artificial 5'UTR Sequences

**Figure 5:**
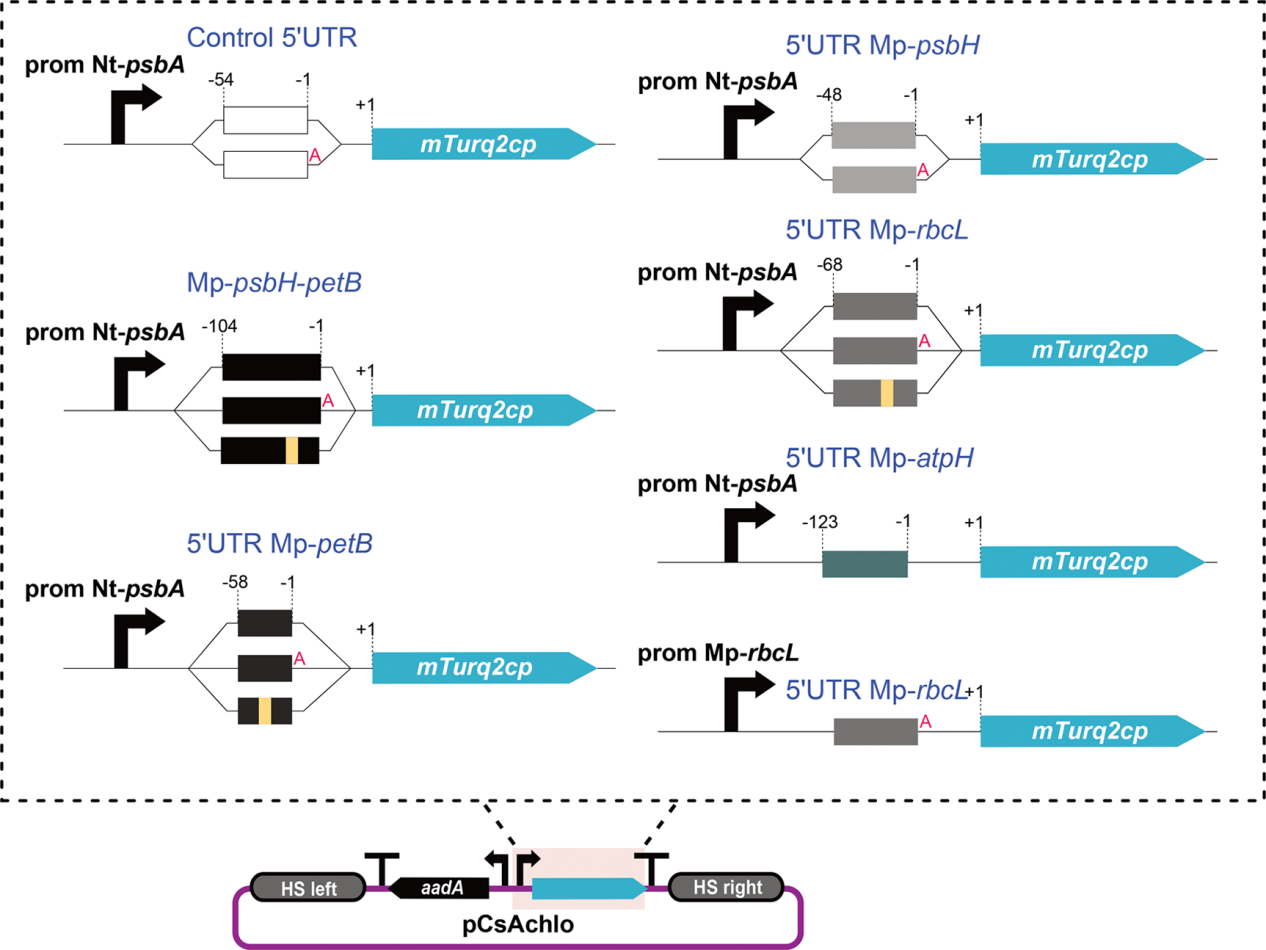
Schematic representation of different constructs. Top: Boxes represent the 5′UTR used. Numbers above the boxes correspond to the nucleotide position in relation to the CDS first nucleotide. Red “A” indicates the extra adenine nucleotide introduced by the common syntax. We cloned the region between Mp-*psbH* and Mp-*petB* (Mp-*psbH*-*petB*, 104 bp in length), 58 bp upstream of the Mp-*petB*, 48 bp upstream of *psbH*, 68 bp upstream of Mp-*rbcL*, and 123 bp upstream of Mp-*atpH*. The amplified sequences were then fused with the Nt-*psbA* promoter (61 bp) and the mTurq2cp fluorescent protein coding sequence. The promoter and 5′UTR (185 bp) of Mp-*rbcL* was also fused to mTurq2cp. All constructs were generated using the OpenPlant kit and Loop assembly. Bottom: Schematic representation of a L2 Loop construct to express the chloroplast codon optimized mTurq2cp fluorescent protein under the control of the tobacco Nt-*psbA* promoter and different combinations of PPR binding sequences (top figure) using the left and right homologous sequences for integration in the chloroplast *rbcL–trnR* intergenic region.^7^

We used the OpenPlant kit^7^ for the generation of the constructs. More specifically, we cloned, as 5′UTR DNA parts, the intergenic region between the *MarchantiapsbH* and *petB* genes (104 bp in length) and sequences corresponding to the 5′UTRs of the *petB* gene (58 bp), *rbcL* (68 bp), *atpH* (123 bp), and *psbH* (48 bp). The amplified sequences were then fused downstream of the tobacco (*Nicotiana tabacum*) *psbA* promoter (61 bp). The intact Nt-*psbA* promoter has been reported to have activity in *Marchantia*, albeit with low expression levels.^4^ The hybrid promoter elements were assembled with the mTurq2cp^4^ fluorescent protein reporter (Figure 5 and Figure S5a).

Chloroplast protein synthesis is mediated by bacterial-type 70S ribosomes, and translation initiation is mediated by ribosome-binding sites, adjacent to the start codon on an mRNA. The sequence and spacing between the ribosome-binding sequence and the start codon is known to be important for the efficiency of translation initiation in cyanobacteria and chloroplasts.^46^ The default common syntax for Type IIS assembly DNA parts^47^ introduces extra sequences at the termini of each element. The assembly of a 5′UTR part can introduce an extra adenosine (A) nucleotide upstream of the ATG start codon. To test whether this has an effect on the expression efficiency of the transgene in *Marchantia* chloroplasts we generated two versions of the constructs, a version for standard assembly with an extra “A” and customized versions without. For the latter, we generated new L0 5'UTR parts with ATGg as the 3′ overhang and mTurq2cp L0 constructs with ATGg as the 5′ overhang. We also generated constructs with mutant PPR binding sites, which contained sequence changes in the putative PPR protein binding site (Figure 5 and Figure S5a). As an additional control, we used a construct with the Nt-*psbA* core promoter fused to a 54 bp sequence containing the multicloning site from the pUC18 vector (45 bp) and a synthetic ribosome binding sequence^48^ (hereafter called “control 5′UTR”). Transplastomic plants containing this construct showed very low levels of fluorescence.^7^

The modified genes were assembled in chloroplast transformation vectors that contained the *aadA* spectinomycin resistance gene and flanking sequences for insertion by homologous recombination into the *rbcL-trnR* intergenic region of the *Marchantia* plastid genome. Chloroplasts were transformed by particle bombardment of germinating *Marchantia* spores, which are relatively easy to harvest in large numbers after sexual crossing, and can be stored indefinitely in a cold, desiccated state before use. DNAdel (Seashell Technology) nanoparticles were used as plasmid DNA carriers for the biolistic delivery into chloroplasts. The use of DNAdel reduces the time and labor required for loading of the plasmid DNA onto the microcarrier used for DNA delivery, compared to conventional metal carriers.

Three weeks after bombardment successful transformants were visible under a fluorescence stereomicroscope. After six–eight weeks on antibiotic selection, plants were tested for homoplasticity (Figure S5b,c). Five independent homoplastic lines for each construct were obtained. Little variation in levels of fluorescence was seen between the independent homoplastic lines, when examined using a stereo fluorescent microscope. Plants transformed with the 5′UTR Mp*-psbH* exhibited similar levels of expression to the control 5′UTR and were not further characterized (Figure S6).

### Testing the artificial 5'UTR Sequences

**Figure 6:**
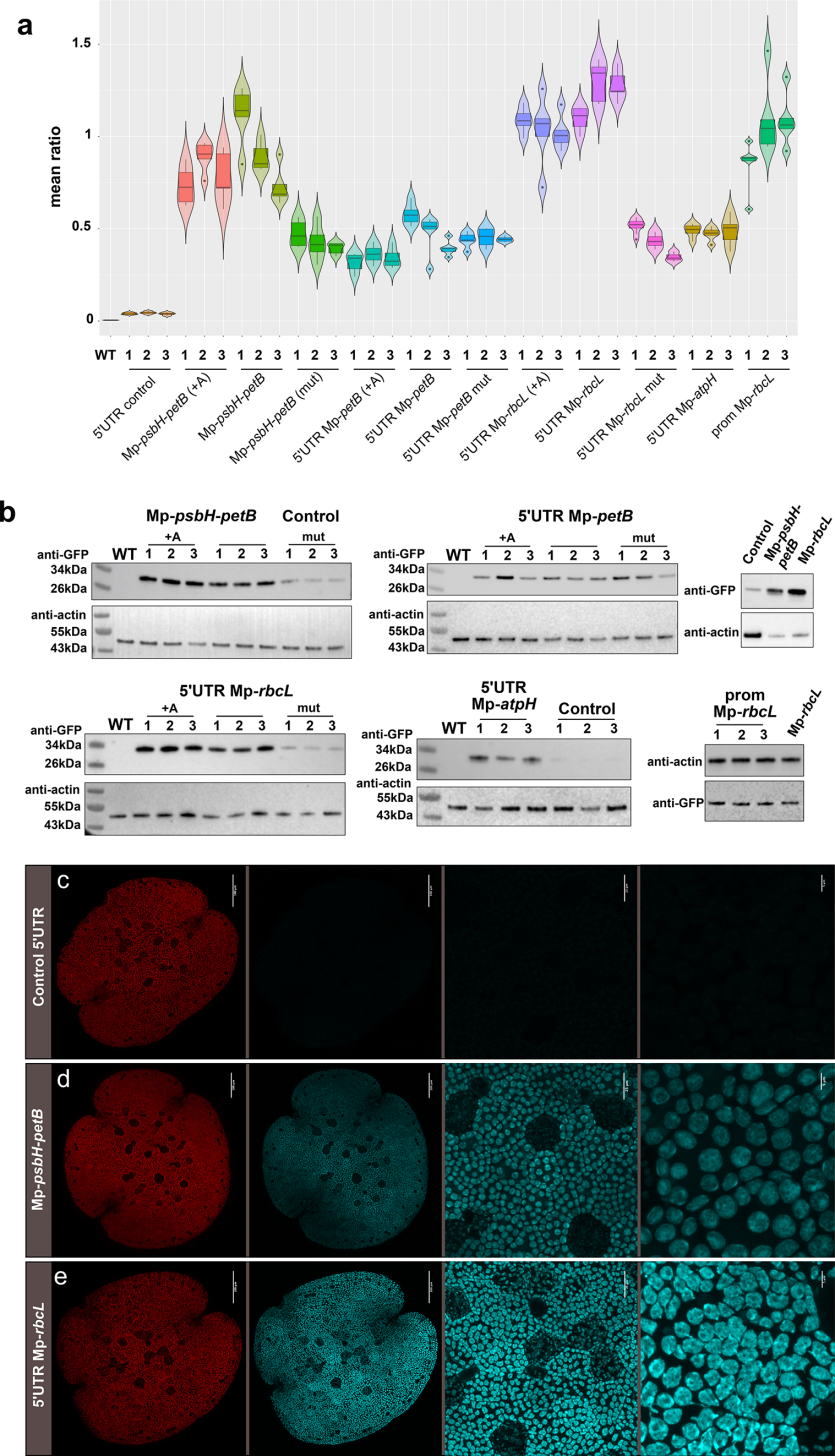
Foreign protein accumulation in transplastomic lines harboring various candidate stabilization elements. (a) Mean ratio of cyan and chlorophyll fluorescence. Five gemmae per line, for three lines per construct, were imaged and the ratio of cyan to chlorophyll fluorescence was calculated. 5′UTR Mp-*rbcL* confers the highest levels of expression followed by Mp-*psbH*-*petB*. 5′UTR Mp*-petB* and 5′UTR Mp-*atpH* have similar levels of expression. Expression levels are reduced for both when the predicted PPR binding sequence is mutated. The addition of an adenine between the 5′UTR and the mTurq2cp coding sequence does not significantly affect the expression of mTurq2cp . (b) Western blots. Immunoblot analysis of mTurq2cp accumulation in transplastomic lines. Total cellular proteins were separated by denaturing gel electrophoresis, blotted and probed with anti-GFP and antiactin antibodies. +A: Adenine introduced by the common syntax present between the 5′UTR and the mTurq2cp coding sequence, Mut: predicted PPR binding sequence mutated. Numbers correspond to three independent lines per construct used. (c–e) Microscopy images of *Marchantia* transplastomic 0-day gemmae expressing the mTurq2cp fluorescent protein under the control of the Nt-*psbA* promoter fused to different candidate stabilization sequences: control 5′UTR, Mp-*psbH*-*petB*, and 5′UTR Mp-*rbcL*. From left to right, first panel: chlorophyll autofluorescence channel (Scale bars: 100 μm), second panel: mTurq2cp channel (Scale bars: 100 μm), third and fourth panel: higher magnification images showing mTurq2cp accumulation inside the chloroplasts of all cells (scale bars: 20 and 5 μm, respectively). All images acquired using identical instrument settings. 5′UTR Mp-*rbcL* confers the highest levels of expression followed by Mp-*psbH*-*petB*.

Three independent homoplastic lines for each construct were selected for further investigation. We developed and applied a three-step image processing pipeline to quantify chloroplast fluorescence intensity. This consisted of (i) acquisition of two-channel fluorescent micrographs using a confocal microscope, with a blue channel tuned to capture cyan fluorescent protein (CFP) fluorescence and a red channel tuned for chlorophyll autofluorescence, (ii) automated segmentation using a custom Fiji macro to identify regions of interest (ROI), and (iii) quantification of fluorescence intensity levels in each channel within each ROI. Mean CFP fluorescence intensity within each ROI was normalized by chlorophyll autofluorescence to account for fluorescence signal attenuation for plastids deeper within the sample^49^ (Figure S6 and Figure S7). First we report the results from transformants containing custom 5′UTR parts with native sequence and spacing adjacent to the start codon of the reporter gene. The highest levels of mTurq2cp fluorescence were measured in plants transformed with constructs containing the 5′UTR Mp-*rbcL* sequence (Figure 6a and Table S7). Plants transformed with constructs containing mutations in the putative MRL1 PPR binding site within the 5′UTR Mp*-rbcL* sequence showed a reduction, but not complete loss of fluorescent protein levels. This is not unexpected since the ribosome binding sequence and promoter were still present.

The Mp*-psbH*-*petB* intergenic region also conferred high levels of fluorescence, although levels were lower than those of plants containing the 5′UTR Mp-*rbcL* sequence. Plants transformed with constructs containing a 10 bp mutation in the putative HCF152 PPR binding site (Table S6) in the Mp-*psbH*-*petB* sequence showed reduced fluorescence levels. The 5′UTR Mp-*petB* sequence also conferred higher levels of fluorescence protein expression compared to the control 5′UTR but lower than that of the Mp-*psbH*-*petB* intergenic region. Plants transformed with constructs containing the 5′UTR Mp-*petB* sequence with a 15 bp mutation that removed the putative binding site for HCF152 did not show significant reduction in fluorescence (Table S6). The 5′UTR Mp-*atpH* sequence produced levels of mTurq2cp fluorescence similar to that of 5′UTR Mp-*petB*.

The standardized syntax for Type IIS assembly of plant genes contains a site for gene fusions at the ATG initiation codon, which requires the sequence AATG to be placed at the junction of 5′UTR and coding sequence. We also tested the activity of constructs assembled this way, bearing an additional A residue adjacent to the start codon, in order to determine any effects on the efficiency of gene expression. Fluorescence levels were only slightly lowered compared to plants transformed with constructs containing the 5′UTR-(ATGg) sequences, indicating that the extra “A” introduced by the common syntax overhang did not have major effects on expression of the marker transgene. These observations were further supported by Western blot studies of fluorescent protein levels in the plants.

Detergent soluble proteins were extracted from three independent lines for each construct, and fractionated by SDS polyacrylamide gel electrophoresis. mTurq2cp protein levels were assayed by Western blotting using an anti-GFP antibody, and an antiactin antibody was used to measure levels of endogenous actin protein as a loading control (Figure 6b). Consistent with the results obtained using ratiometric imaging, the 5′UTR Mp-*rbcL* leader sequence conferred the highest levels of protein accumulation followed by the Mp-*psbH-petB* intergenic region. Constructs containing 5′UTR Mp-*petB* and 5′UTR Mp-*atpH* showed similar, lower levels of expression. The addition of an extra “A” between the 5′UTR and the mTurq2cp coding sequence did not greatly affect the expression of mTurq2cp in these experiments. However, substantially lower levels of fluorescent protein were seen in plants bearing mutations in the predicted PPR binding sites in 5′UTRs derived from Mp-*rbcL* and the Mp-*psbH*-*petB* intergenic region.

On the basis of these analyses the mRNA leader sequences corresponding to the Mp*-psbH*-*petB* intergenic region and 5′UTR Mp-*rbcL* were selected as the best candidates for generating high level gene expression in *Marchantia* chloroplasts. (Figure 6c–e).

### *Marchantia rbcL* Native Promoter

The selected mRNA leader sequences with PPR-binding sites were all tested with the *N. tabacumpsbA* promoter. To test the importance of the promoter in driving transgene expression, we cloned the entire promoter and 5′UTR from the Mp-*rbcL* gene (185 bp upstream of the start codon), in order to compare it with the Nt-*psbA* promoter-driven version. Native transcripts from the Mp-*rbcL* promoter were found to accumulate at notably high levels in *Marchantia* (Figure 3c). The native promoter was fused to the mTurq2cp coding sequence, and transformed into the *Marchantia* chloroplast genome as described for the other gene fusions. Confocal microscopy of the transformed plants confirmed (i) the exclusive chloroplast localization of the expressed transgene, and (ii) high levels of fluorescent protein expression. High levels of mTurq2cp protein accumulation were further confirmed by ratiometric fluorescence measurements and a Western blot analysis. However, the levels were not significantly over those conferred by the Nt-*psbA* promoter fusion (Figure 6a and Figure S6). This indicated that either both promoters had similar properties in *Marchantia* chloroplasts, or that rates of RNA transcription, mRNA stability, translation or protein stability might be saturated, and rate limiting.

### Quantification of Transgene Expression

**Figure 7:**
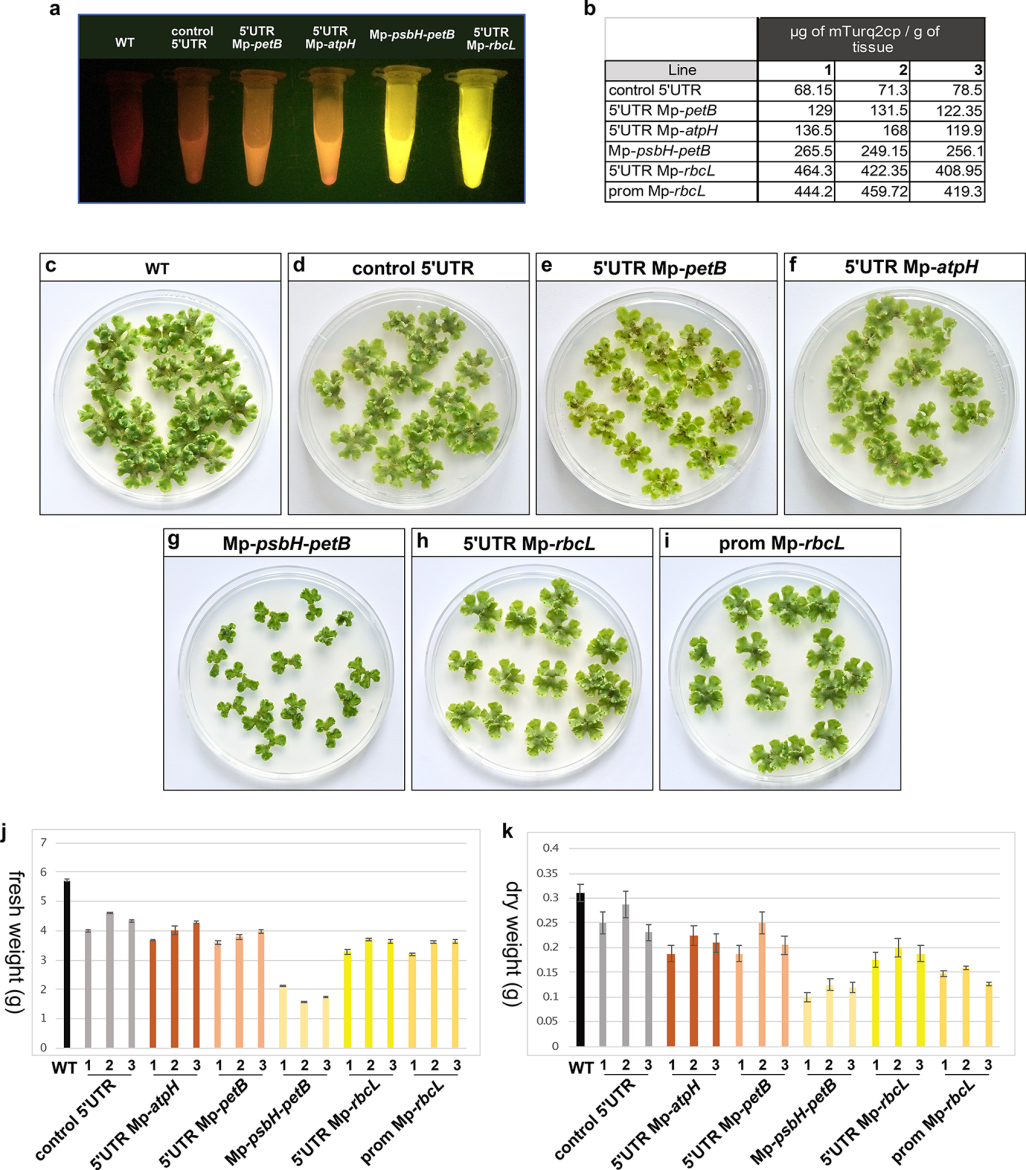
(a) Total protein extract from 200 mg of 1 month old gemmae under blue light transillumination. Red corresponds to chlorophyll autofluorescence and yellow to mTurq2cp fluorescence. Extract from plants transformed with the construct containing 5′UTR Mp-*rbcL* are exhibiting the brightest fluorescence. (b) Estimation of μg of mTurq2cp /g of fresh tissue, for three independent lines per construct. (c–i) Comparison of growth between wild type and transplastomic *Marchantia* one month old gemmae expressing different constructs. All transplastomic plants showed a reduction in growth and biomass. Plants transformed with the construct containing the Mp-*psbH*-*petB* sequence showed the most extreme growth reduction phenotype. (j,k) The fresh and dry weight was measured for 30 one month old gemmae, and the average values of two different experiments are shown on graphs j and k. Plants transformed with the construct containing the 5′UTR Mp-*rbcL*, even though they express the highest levels of mTurq2cp , only showed an approximately 35% reduction in biomass. Error bars: standard error.

In order to estimate the amount of protein produced in transplastomic *Marchantia* plants we expressed His6-tagged mTurquoise2 in *E. coli* under the control of the T7 promoter and purified the protein by affinity chromatography (Figure S8). Serial dilutions of the purified mTurquoise2 were used to create a standard curve (fluorescence emission *versus* protein concentration) to allow accurate measurement of protein levels. Total protein was extracted from the *Marchantia* thallus tissue of plants harboring different constructs (see Materials). The CFP fluorescence for each sample was then measured using a Clariostar plate reader and the protein concentration was calculated based on the standard curve. Up to 460 μg per g of tissue (∼15% total soluble protein) was obtained from homoplastic plants harboring the construct containing the Nt-*psbA* promoter and 5′UTR Mp-*rbcL* sequence (Figure 7a, b).

### Growth Rates of Transplastomic Plants

Growth defects have been observed in plant species with high levels of chloroplast transgene expression.^50,51^ Very high levels of expression of a stable protein can lead to delayed plant growth.^2^ To test whether the accumulation of foreign proteins had an effect on *Marchantia* growth, we compared the growth of wild-type gemmae with those of lines transformed with the different constructs (Figure 7c-k). The accumulation of fresh and dry weight was measured after one month of growth on agar-based media. Plants transformed with the construct containing the 5′UTR Mp-*rbcL*, which resulted in the highest levels of mTurq2cp accumulation, showed an approximately 35% biomass reduction compared to wild type. Interestingly, in comparison to other systems, *Marchantia* showed a higher tolerance to foreign protein accumulation in the chloroplast. For example, the potato showed significant biomass decrease in response to green fluorescence protein (GFP) overexpression.^21^ Interestingly, plants transformed with the construct containing the 5′UTR Mp-*rbcL* construct showed lower size reduction than those transformed with the Mp-*psbH*-*petB* containing construct, despite higher levels of transgene accumulation.

## Conclusions

Chloroplasts are attractive vehicles for transgene hyperexpression. Chloroplasts are sites for energy generation and high-level protein expression and play a major role in metabolite production in plant cells. Plastid genes are present in high copy numbers per cell, can be highly transcribed, and are not subject to gene silencing. The plastid genome is compact and conserved across the terrestrial plants, and shows great promise as a platform for low-cost, large scale bioproduction.

Recent work in the field has demonstrated the requirement for proper post-transcriptional regulation for high level gene expression in the chloroplasts of angiosperm plants.^9,12,37,44^ In particular, nuclear-encoded PPR-proteins play a direct role in stabilizing the termini of specific mRNAs by direct binding, likely to protect the mRNAs against exoribonuclease degradation. The plastid genomes of early divergent plants, like *Marchantia*, possess a coding capacity broadly similar to gymnosperm and angiosperm species. However, gene regulation is different in a number of respects, such as the absence of RNA editing in *Marchantia*. Further, noncoding sequences in the plastid genome have diverged markedly. These key determinants of expression levels remain inaccessible to genetic manipulation, owing to insufficient understanding of native regulation and very limited availability of characterized parts. In order to fully exploit the potential benefits of the *Marchantia* system, we needed to “domesticate” important regulatory functions that allow properly regulated and high-level gene expression.

In this work, we describe the mapping of transcription patterns on the plastid genome of light-grown *Marchantia*. This allowed us to obtain empirical evidence for levels of transcription across the plastid genome. We precisely identified the promoter start sites for a number of highly expressed chloroplast genes. These genes have homologues in better-studied model systems, like tobacco, maize and *Arabidopsis*, where terminal sites for PPR protein binding to mRNAs have been characterized recently. However, sequence drift and the limited size of these functionally important sequences make them difficult to identify by inspection in widely divergent species.

While thousands of gymnosperm and angiosperm plastid genomes are available to build phylogenetic comparisons, the record for bryophytes has been sparse. We have engaged in a program of plastid genome sequencing to expand the data available for liverworts, hornworts and mosses. We have contributed 30 new bryophyte plastid genome sequences, and here, have used the newly expanded set to draw phylogenetic comparisons across the 5′ noncoding sequences of high abundance *Marchantia* transcripts. These regions correspond to mRNA termini that contain PPR protein binding sites in well-characterized angiosperm model systems. These fine-detail comparisons revealed conserved nucleotide sequences that may correspond to binding sites in *Marchantia*, and reflect an ancient origin for PPR-mediated control of gene expression in chloroplasts.

The identification of these conserved domains, which are putative PPR protein binding elements in the 5′UTRs of chloroplast mRNAs, has allowed us to assemble a modular library of DNA parts that could confer transcript stability. In order to test the function of these novel 5′UTR elements, the candidate sequences were each assembled as components of gene fusions between a chosen promoter and the mTurq2cp fluorescent protein coding sequence and terminator. The novel DNA parts were incorporated into chloroplast transformation vectors, and homoplastic transformants were generated. The levels of fluorescent protein expression in transformed plants were measured by microscopy-based ratiometric imaging, Western blot analysis and protein extraction and quantitation. The presence of putative PPR protein binding sites at the 5′ termini of artificial mRNAs conferred markedly higher levels of reporter gene expression. Mutations within the putative binding domains reduced levels of gene expression. Highest levels of gene expression were seen in plants with reporter genes containing active promoters and the 5′UTRs of Mp-*rbcL* and the Mp*-psbH*-*petB* intergenic region. A single inserted gene of interest could produce up to 15% of total soluble protein. Analysis of the growth rates of these plants showed that there was some penalty for hyperexpression in the form of slower growth. Lowered growth rates did not correspond directly to the level of ectopically expressed fluorescent protein, and it is possible that the mRNA transcripts themselves may interfere with growth, perhaps through competition with native transcripts for the different target PPR proteins. This indicates that conditional expression may be useful, through regulation of transcription in the chloroplast, regulation of mRNA stability through conditional expression of heterologous PPR proteins or supplementary expression of any limiting PPR proteins.

The identification and domestication of these mRNA stabilizing elements allows the prospect of enhanced gene design for engineering of the *Marchantia* plastid genome, to take advantage of the speed of this experimental system. Both the hybrid Nt*-psbA* promoter and 5'UTR Mp*-rbcL* and native Mp*-rbcL* promoter-5′UTR sequences show high activity with minimal deleterious effects on growth, and look promising for future work in *Marchantia*. The transformation, regeneration and rescue of homoplastic transformants in tobacco may take 6–9 months, while a similar experiment can take eight weeks in *Marchantia*. Further, the vegetative life cycle for *Marchantia* can take as little as two weeks, and a single cycle through the sexual phase will give rise to millions of progeny as spores. *Marchantia* can grow quickly and it may be useful as a cheap, easy to maintain, and high yielding platform for small-scale bioproduction. Further, the DNA toolkit developed and characterized in *Marchantia* may function in plastids from a wide variety of plants.

## Materials and Methods

### Chloroplast Isolation

Chloroplast isolation buffer (CIB) composition: 50 mM HEPES-KOH pH 7.5, 0.33 M sorbitol, 1 mM MgCl2, 1 mM MnCl_2_, 2 mM EDTA. 5 mM Na-ascorbate and 1% (w/v) BSA (final concentration) were added immediately before use. Percoll (#17-0891-02, GE Healthcare) gradients were prepared as follows: 20 mL 30% (v/v) Percoll solution was prepared by mixing 6 mL Percoll and 14 mL CIB. 10 mL 70% (v/v) Percoll solution was prepared by mixing 7 mL Percoll and 3 mL CIB. For the preparation of 30%:70% (v/v) Percoll gradient, 15 mL of 30% (v/v) Percoll were placed into a 50 mL Falcon tube and 6 mL of 70% (v/v) Percoll solution was carefully underlaid using a 5 mL Gilson pipet.

Plants were grown in a 12 h light:12 h dark cycle, and thallus tissue was harvested 2–3 h after the start of the light cycle to minimize the amount of starch accumulated in chloroplasts. 40 g of tissue was split into four equal parts and each was homogenized using a mortar and pestle in 100 mL of CIB. The homogenate was filtered through two layers of Miracloth (#475855, Millipore) into six 50 mL Falcon tubes and centrifuged at 1200*g* for 7 min. The supernatant was discarded and the pellet from each tube was carefully resuspended in 2 mL of CIB using a paint brush. The resuspended pellet was transferred to the top of a Percoll gradient using a cutoff 1 mL pipet tip, and spun at 7000*g* for 17 min at 4 °C using slow acceleration and deceleration. Broken chloroplasts resided in the top fraction, while intact chloroplasts accumulated at the interface of the two Percoll layers. Chloroplasts from the interface were transferred to a 50 mL falcon tube. 25 mL of CIB was added, and tubes were centrifuged at 1500*g* for 5 min at 4 °C. The supernatant was discarded and the pellet was flash frozen in liquid N_2_.

### RNA Extraction

RNA extraction was performed using the *mir*Vana miRNA Isolation Kit (#AM1560, ThermoFisher/Ambion) according to manufacturer instructions. After RNA extraction, samples were treated with DNase I using the TURBO DNA-*free* Kit (#AM1907, ThermoFisher/Ambion) following the manufacturer’s instructions. The integrity of the DNase treated RNA was confirmed by capillary electrophoresis using the Agilent Bioanalyzer and the Agilent RNA 6000 Nano kit (#5067-1511, Agilent) according to the manufacturer’s instructions.

### Differential RNA-Sequencing

Samples were treated and sequenced by vertis Biotechnologie AG, Germany. Detailed protocol in Figure S2.

### Differential RNA-Sequencing Processing

FASTQ read files were mapped against the Cam-1/2 plastid assembly (Genbank accession no. MH635409) using STAR-2.7.3a.^52^ First, we generated a STAR index for the MH635409 assembly using the FASTA file of the assembly and existing genome annotation in GTF format (with settings as follows: −runMode genomeGenerate −sjdbOverhang 74 −genomeSAindexNbases 7). We then used multisample two pass mapping. In the first pass, samples were pooled and jointly mapped against the index to enable detection of unannotated transcripts and splice junctions. We supplied the genome annotation at this step and used conservative filtering of potential novel splice sites, (with settings as follows: −alignIntronMax 800 −outSJfilterCountUniqueMin 40 40 40 40 −outSJfilterCountTotalMin 50 50 50 50 −sjdbOverhang 74). For the second pass we mapped each library against the index using both the existing genome annotation and the list of novel junctions generated by the first pass, using the same parameters as before. Mapping statistics for each library are provided in Table S1.

We split the SAM output files into reverse and forward mapped reads using samtools view^53^ and converted them to BAM format. Each file was sorted using samtools sort and per base coverage calculated using samtools depth. Base coverage was normalized and expressed as coverage per million mapped reads for each library. Coverage, data processing, and visualization was performed in R version 3.5.1. Plots were generated using ggplot2, ggbio^54^ and circlize^55^ packages.

Gene expression was quantified using kallisto.^56^ Protein coding transcript sequences were extracted from the MH635409 assembly sequence and used to build a kallisto index. FASTQ files from control libraries were processed using kallisto quant. Levels of gene expression were reported in units of transcripts per million (TPM).

### TSS Identification

A 5′ end was annotated as a TSS when it had the following: (i) a coverage in both TEX+/TEX– libraries of at least >2 per million mapped reads, (ii) a start at the same genomic position (nucleotide) in both libraries, and (iii) an enrichment >1 in the TEX+ library (109 putative TSS in total). A 5′ end that was not enriched in TEX+ libraries was accepted as a TSS if it extended into an annotated gene (65 putative TSSs in total). We assigned 12 additional TSS that do not fall into the above categories when they extended into an annotated gene and a PEP promoter motif was predicted using MEME.^31^

### *Marchantia* PPR Homologue Prediction

Orthofinder was used^45^ for the identification of PPR and HAT homologues between *M. polymorpha* and *A. thaliana* and maize.

### DNA Extraction, Sequencing, and *De Novo* Bryophyte Plastid Genome Assemblies

DNA extraction, sequencing and *de novo* assembly of plastid genomes were performed according to the literature.^36^ In addition, NGS data generated for a previous study^36,57^ were used for *de novo* assembly of *Anomodon attenuates*, *Atrichum angustatum*, *Bartramia pomiformis*, *Bryum argenteum*, *Entosthodon attenuates*, *Funaria hygrometrica*, *Hypnum imponens*, *Orthotrichum stellatum*, *Ptychomnion cygnisetum*, *Sphagnum palustre*, *Tetraphis pellucida*, and *Ulota hutchinsiae* moss plastid genome sequences. Assemblies were performed using GetOrganelle^58^ (Table S5) and annotated using GeSeq.^59^ Genome alignments were performed using MUSCLE.^60^ All new plastid genome assemblies are available in Genbank.

### *Marchantia* Chloroplast DNA Manipulation

Genomic DNA was extracted according to the literature.^7^ Constructs were generated using DNA parts and vectors from the OpenPlant kit.^7^ Construct sequences are listed in Table S6. Primers used for construct generation are listed in Table S8. Chloroplast transformation was performed as previously described in the literature.^7^ The genotyping of transplastomic lines was performed as previously described in the literature.^7^ Genotyping primers used are listed in Table S8. All new DNA parts are available from Addgene.

### Imaging

Gemmae were plated on half strength Gamborg B5 plus vitamins (#G0210, Duchefa Biochemie) with 1.2% (w/v) agar plates and placed in a growth cabinet for 3 days under continuous light with 150 μE m^–2^ s^–1^ light intensity at 21 °C. A gene frame (#AB0576, ThermoFisher) was positioned on a glass slide and 30 μL of half strength Gamborg B5 1.2% (w/v) agar placed within the gene frame. Five gemmae were then placed within the media filled gene frame, 30 μL of Milli-Q water was added, and then a coverslip was used to seal the geneframe. Plants were then imaged immediately using an SP8 fluorescent confocal microscope. All images were acquired using the same instrument setting, Cyan and chlorophyll. Sixteen Z stacks, 3 μm thickness.

Images were acquired on an upright Leica SP8X confocal microscope equipped with a 460–670 nm supercontinuum white light laser, 2 CW laser lines 405 nm, and 442 nm, and 5 channel spectral scanhead (4 hybrid detectors and 1 PMT). Imaging was conducted using either a 20× air objective (HC PL APO 20×/0.75 CS2) or a 40× water immersion objective (HC PL APO 40×/1.10 W CORR CS2). Excitation laser wavelength and captured emitted fluorescence wavelength window were as follows: for mTurq2cp (442 nm, 460–485 nm) and for chlorophyll autofluorescence (488 or 515, 670–700 nm). Chlorophyll autofluorescence was imaged simultaneously with mTurq2cp.

### Plastid Segmentation Pipeline

Plastid segmentation was achieved using an automated Fiji macro as described previously,^49^ the source code is included in Figure S7c. In brief, the chlorophyll autofluorescence channel was duplicated, and the new copy subjected to a series of smoothing and thresholding steps using the Phansalkar algorithm,^61^ and the subsequent segmented regions were split using a watershed algorithm. Regions of interest were then used for quantification of marker gene and chlorophyll fluorescence and analysis of plastid parameters such as size and shape. Analysis in Figure 6 is based on the average fluorescence intensity within each ROI, with the CFP channel normalized by the chlorophyll channel. The full data set (including additional parameters such as maximum and minimum fluorescence intensity within each ROI as well as area of ROIs) is included as Table S7.

### Western Blotting

*Marchantia* thallus tissue (100 mg) was excised from plants grown for 4 weeks on half strength Gamborg B5 medium including vitamins with 1.2% (w/v) agar, at 21 °C in continuous light, 150 μE m^–2^ s^–1^) and ground in liquid nitrogen. The tissue powder was resuspended in 500 μL 5× Laemmli loading buffer (0.2 M Tris-Hcl pH 6.8, 5% (w/v) SDS, 25% (v/v) glycerol, 0.25 M DTT, 0.05% (w/v) bromophenol blue) with added Roche cOmplete protease inhibitor (#11836170001, Roche). Samples were further diluted 21 times in 5× Laemmli loading buffer containing Roche protease inhibitor, heated at 95 °C for 5 min and centrifuged at 10 000*g* for 10 min. The supernatant was transferred to a new tube. Equal amounts of proteins were separated by denaturing electrophoresis in NuPAGE gel (#NP0322BOX, Invitrogen) and electrotransferred to nitrocellulose membranes using the iBlot2 Dry Blotting System (ThermoFisher). mTurq2cp was immunodetected with anti-GFP antibody (1:4000 dilution) (JL-8, #632380, Takara) and antimouse-HRP (1:15000 dilution) (#A9044, Sigma) antibodies. Actin was immunodetected with antiactin (plant) (1:1500 dilution) (#A0480, Sigma) and (1:15000 dilution) antimouse-HRP (#A9044, Sigma) antibodies, using the iBind Western Starter Kit (#SLF1000S, ThermoFisher). Western blots were visualized using the ECL Select Western Blotting Detection Reagent (#GERPN2235, GE) following the manufacturer’s instructions. Images were acquired using a Syngene Gel Documentation system G:BOX F3.

### Plant Biomass Estimation

For each line 30 gemmae were placed on two Petri dishes with 25 mL of media (half strength Gamborg B5 plus vitamins) and grown for a month, at 21 °C, with continuous light, 150 μE m^–2^ s^–1^. The fresh and dry weight was measured using a scale.

### Total Soluble Protein Estimation

*Marchantia* thallus tissue (200 mg) from 4 week old plants grown on half strength Gamborg B5 medium including vitamins and 1.2% (w/v) agar, at 21 °C in continuous light, 150 μE m^–2^ s^–1^ was ground in liquid nitrogen and resuspended in 700 μL protein extraction buffer (50 mM Tris-HCl pH 7.5, 150 mM NaCl, TWEEN 20 0.1% (v/v), 10% (v/v) glycerol, 1 mM DTT) plus Roche cOmplete protease inhibitor (# 11836170001, Roche). Total soluble protein concentration was estimated using a Pierce 660 nm Protein Assay Kit as above (#22662, Thermo Scientific).

### Protein Yield Estimation

*E. coli* BL21 Star (DE3) (#C601003, Invitrogen) was transformed with the pCRB SREI6His plasmid^4^ to express the mTurquoise2 protein. A culture of 10 mL was used to inoculate 250 mL of LB medium supplemented with ampicillin and grown in 2.5 L baffled Tunair shake flasks (#Z710822, Sigma-Aldrich) at 37 °C with vigorous shaking (200 rpm). Cultures were monitored by spectrophotometry until OD_600_ reached 0.6. T7 RNA polymerase expression was induced by the addition of IPTG to a final concentration of 1 mM. Cultures were grown for 5 h at 30 °C, with shaking at 200 rpm. Cells were then harvested by centrifugation at 5000*g* for 12 min at 4 °C. To purify the recombinant protein under native conditions, the pellet was processed using the Ni-NTA Fast Start Kit (#30600, Qiagen), and cells were disrupted by lysozyme and detergent treatment according to the manufacturer’s instructions. Purified protein was concentrated using an Amicon Filter 3K (#UFC500324, Millipore). In order to avoid any interference with downstream procedures, imidazole was removed using a Zeba spin desalting column (#89882, Thermo Scientific) following the manufacturer’s protocol. Purified protein was stored in 50 mM sodium phosphate, pH 7.4 with 5 mM benzamidine at −20 °C.

The concentration of the mTurquoise2 protein was determined using a Pierce 660 nm Protein Assay Kit (#22662, Thermo Scientific) and used as reference to build a mTurquoise2 standard curve (linear regression) based on fluorescence (random fluorescence units (RFU)) against concentration.

This curve was employed to estimate mTurq2cp protein amount in *Marchantia* samples (prepared following the same steps described in the total soluble protein estimation) per gram of tissue. Samples values were adjusted by subtracting the fluorescence values of the blank. In all the cases, a CLARIOstar (BMG) plate reader was used with an excitation and emission wavelength appropriate for mTurq2cp measurement (excitation: 430–20 nm, emission: 474–20 nm, gain 500 nm).

## Supporting Information Available

Figure S1, Operon and gene map of the *Marchantia* Cam-1/2 plastid genome; Figure S2, Preparation of ±TEX cDNA libraries for Illumina sequencing; Figure S3, *Marchantia* HCF152, HCF107, MRL1, and PPR10 homologues; Figure S4, Multiple sequence alignments of regions upstream the 5′ UTR of *petB*, *psbH*, *rbcL*, and *atpH* of the 51 bryophyte plastid genomes used in this study; Figure S5, Schematic representation of different constructs, validation of DNA parts and vectors for chloroplast transformation; Figure S6, Microscopy images of *Marchantia* transplastomic gemmae; Figure S7, Schematic of sample preparation and plastid segmentation pipeline; Figure S8, Coomassie blue-stained protein gel of mTurquoise2 recombinant protein; Table S1: Mapping statistics for dRNaseq; Table S4: Bryophyte plastid genomes used in this study; Table S5: Moss genome assemblies overview; Table S8: List of primers used in this study (PDF)
Table S2: List of TSSs identified using dRNaseq; Table S3: List of MEME identified promoter motifs; Table S6: List of constructs (XLSX)
Table S7: Microscopy image quantification (XLSX)
