# Supplementary material for: Construction of DNA Tools for Hyperexpression in Marchantia Chloroplasts
Source: ACS Synth Biol. 2021 Jun 7;10(7):1651–66. doi: 10.1021/acssynbio.0c00637 (PMC8296666; doi:10.1021/acssynbio.0c00637)
Supplement: Supplementary file 1 — sb0c00637_si_001.pdf [file sb0c00637_si_001.pdf]

SUPPLEMENTARY INFORMATION

Construction of DNA Tools for Hyperexpression in *Marchantia* Chloroplasts.

Eftychios Frangedakis[1], Fernando Guzman-Chavez[1], Marius Rebmann[1], Kasey Markel[1,6], Ying Yu[2,9], Artemis Perraki[1,7], Sze Wai Tse[1], Yang Liu[3], Jenna Rever[1], Susanna Sauret-Gueto[1,8], Bernard Goffinet[4], Harald Schneider[5] and Jim Haseloff[1] *

[1] Department of Plant Sciences, University of Cambridge, Downing Street, Cambridge. UK

[2] College of Life and Environmental Sciences, Hangzhou Normal University, Hangzhou 311121, China

[3] Fairy Lake Botanical Garden & Chinese Academy of Sciences, Shenzhen, China

[4] Department of Ecology and Evolutionary Biology, University of Connecticut, Storrs, CT, US

[5] Center for Integrative Conservation, Xishuangbanna Tropical Botanical Garden, Chinese Academy of Sciences, Menglun, Yunnan, China

[6] Present address: Department of Plant Biology, University of California, Davis, US

[7]Present address: Institute of Molecular Biology and Biotechnology, Foundation for Research and Technology - Hellas, Heraklion, Crete, Greece

[8]Present address: Crop Science Centre, University of Cambridge, 93 Lawrence Weaver Road, Cambridge CB3 0LE, UK

[9]Present address: College of Life and Environmental Sciences, Huangshan University, Huangshan 245041, China

*corresponding author email address: jh295@cam.ac.uk

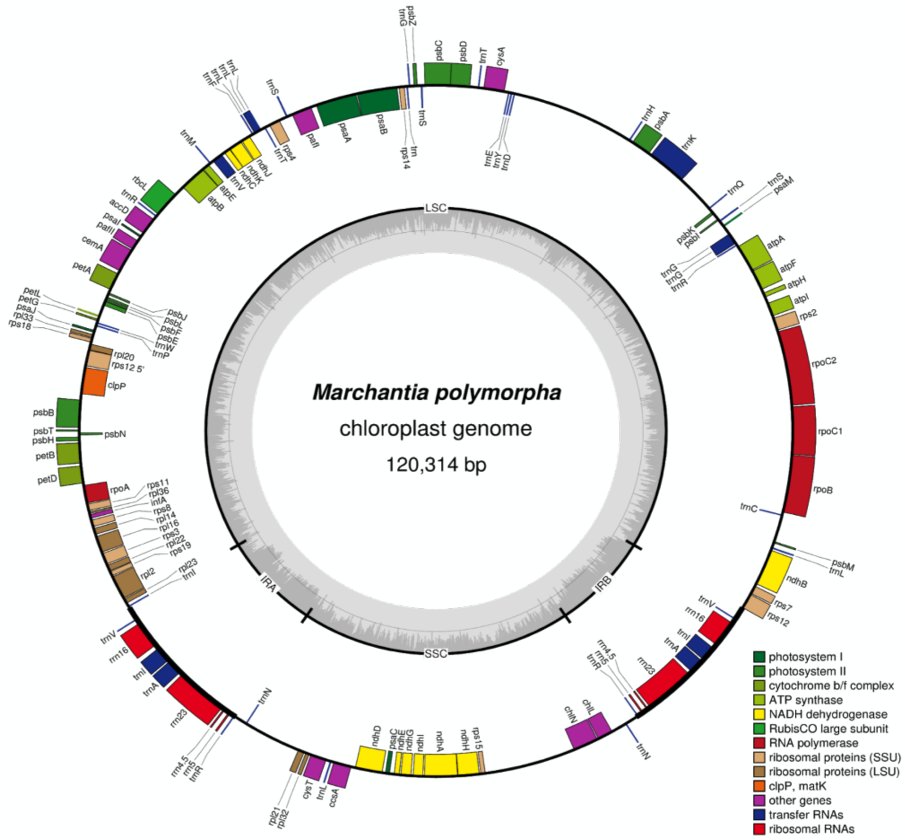
Marchantia polymorpha
chloroplast genome
120,314 bp
LSC
IRA
IRB
SSC
photosystem I
photosystem II
cytochrome b/f complex
ATP synthase
NADH dehydrogenase
RubisCO large subunit
RNA polymerase
ribosomal proteins (SSU)
ribosomal proteins (LSU)
clpP, matK
other genes
transfer RNAs
ribosomal RNAs

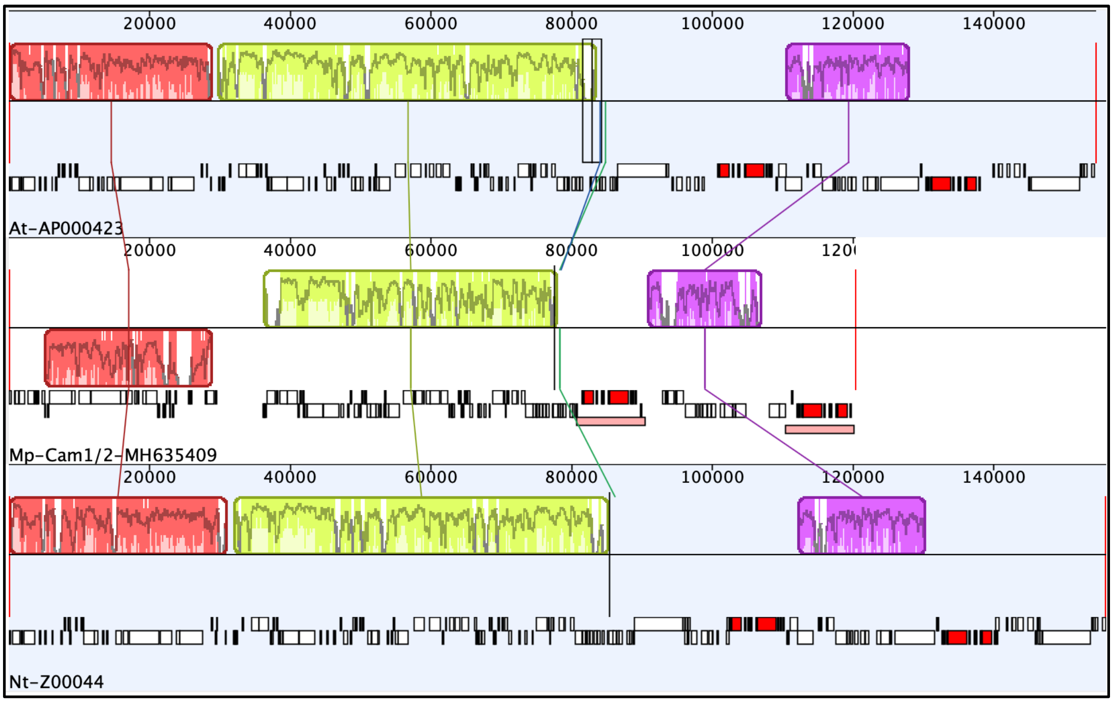
20000
40000
60000
80000
100000
120000
140000
At-AP000423
Mp-Cam1/2-MH635409
Nt-Z00044

Figure S1: Operon and gene map of the *Marchantia* Cam-1/2 plastid genome.
a) The outer circle depicts the gene organization of the *Marchantia* plastid genome (MH635409). The graph was generated using OGDraw [1]. Genes are colour coded based on their function listed at the bottom right of the figure. The Cam-1/2 plastome assembly was validated by comparison to both Sanger sequencing data covering ~10% of the plastome and the newly published Kit-2 plastome (NC_037507.1) assembly [2]. In both cases, validation supports a highly accurate assembly process.

b) Mauve [3] whole plastid genome alignment *Arabidopsis* (AP000423), tobacco (Z00044) and *Marchantia*. The coloured blocks indicate regions of homology between the plastid genomes of three species. Blocks of the same colour, connected with a line, indicate entirely co-linear and homologous regions (gene clusters). The boundaries of coloured blocks indicate potential breakpoints of genome rearrangement, unless sequence has been gained or lost in the breakpoint region.

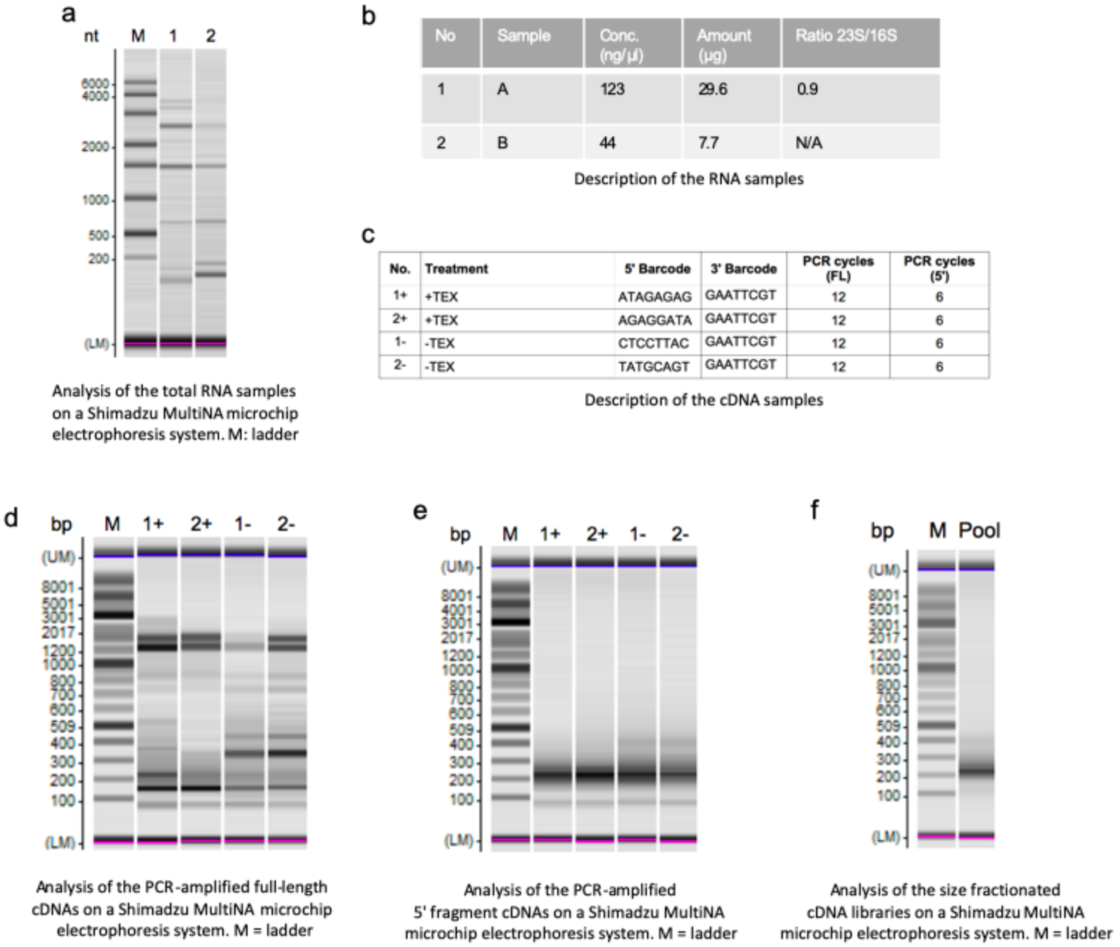

No	Sample	Conc. (ng/µl)	Amount (µg)	Ratio 23S/16S
1	A	123	29.6	0.9
2	B	44	7.7	N/A

No.	Treatment	5' Barcode	3' Barcode	PCR cycles (FL)	PCR cycles (5')
1+	+TEX	ATAGAGAG	GAATTCGT	12	6
2+	+TEX	AGAGGATA	GAATTCGT	12	6
1-	-TEX	CTCCTTAC	GAATTCGT	12	6
2-	-TEX	TATGCAGT	GAATTCGT	12	6

Figure S2: Preparation of +/-TEX cDNA libraries for Illumina sequencing

a-b) Analysis of total RNA

The total RNA samples were examined by capillary electrophoresis and RNA concentration was determined.

c) cDNA synthesis from +/-TEX treated RNA

The total RNA samples were split into two halves and one half was subjected to Terminator exonuclease (TEX) treatment. 2U of TEX enzyme (#TER51020, Lucigen) per 500 ng of RNA were used. Incubation was 1 hour at 30°C. The other half was left untreated (-TEX). The + and -TEX treated RNAs were poly(A)-tailed using poly(A) polymerase. The 5'PPP were converted to 5’P structures using RNA 5' Polyphosphatase (#RP8092H, Epicentre). 5’ Illumina sequencing adaptor was ligated to the 5'P of the +/-TEX treated RNA. First- strand cDNA synthesis was performed using an oligo(dT)-adapter primer and the M-MLV reverse transcriptase. The resulting cDNAs were PCR-amplified to about 10-20 ng/µL using a high-fidelity DNA polymerase (cycle numbers are indicated in the table).

d) The cDNAs were purified using the Agencourt AMPure XP kit (Beckman Coulter Genomics) and were analyzed by capillary electrophoresis

e) For Illumina sequencing, 100 – 300 bp long 5' fragments were isolated from the full-length cDNAs. For this purpose, the cDNA preparations were fragmented and the 5'-cDNA fragments

were then bound to streptavidin magnetic beads. The bound cDNAs were blunted and the 3' Illumina sequencing adapter was ligated to the 3' ends of the cDNA fragments. The bead bound cDNAs were finally PCR-amplified. The PCR cycles performed and the barcode sequences, which are attached to the 5' and 3' ends of the cDNAs, are described in the table at (c).

f) Pool generation and size fractionation

For Illumina NextSeq sequencing, the samples were pooled in approximately equimolar amounts. The library pool was fractionated in the size range of 200-500 bp using a preparative agarose gel. An aliquot of the size fractionated cDNA pool was analyzed by capillary electrophoresis. The cDNAs have a size of about 200 – 500 bp. The primers used for PCR amplification were designed for TruSeq sequencing according to the instructions of Illumina. The following adapter sequences flank the DNA insert:

TruSeq_Sense_primer i5 Barcode 5'-AATGATACGGCGACCACCGAGATCTACAC-NNNNNNNN-ACACTCTTTCCCTACACGACGCTCTTCCGATCT-3’
TruSeq_Antisense_primer i7 Barcode 5’-CAAGCAGAAGACGGCATACGAGAT-NNNNNNNN-GTGACTGGAGTTCAGACGTGTGCTCTTCCGATCT-3’
The combined length of the flanking sequences is 136 bases.

The cDNA pool was single end sequenced on an Illumina NextSeq 500 system using 1x75 bp read length.

Sample A was used for the TSS predictions.
Both Sample A and B were used for the identification of highly expressed genes.

a

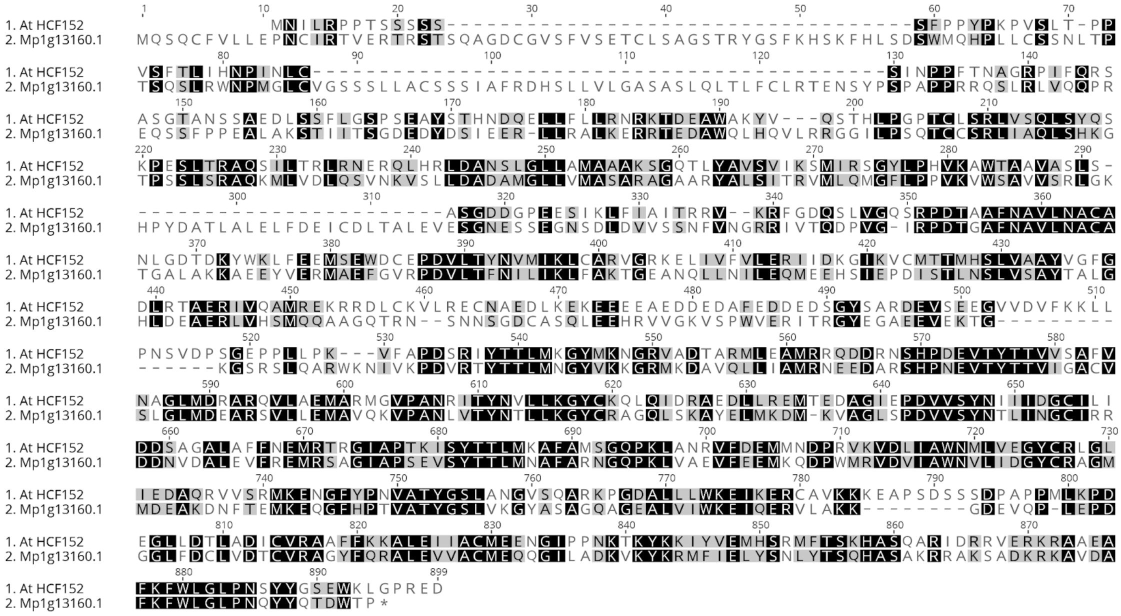

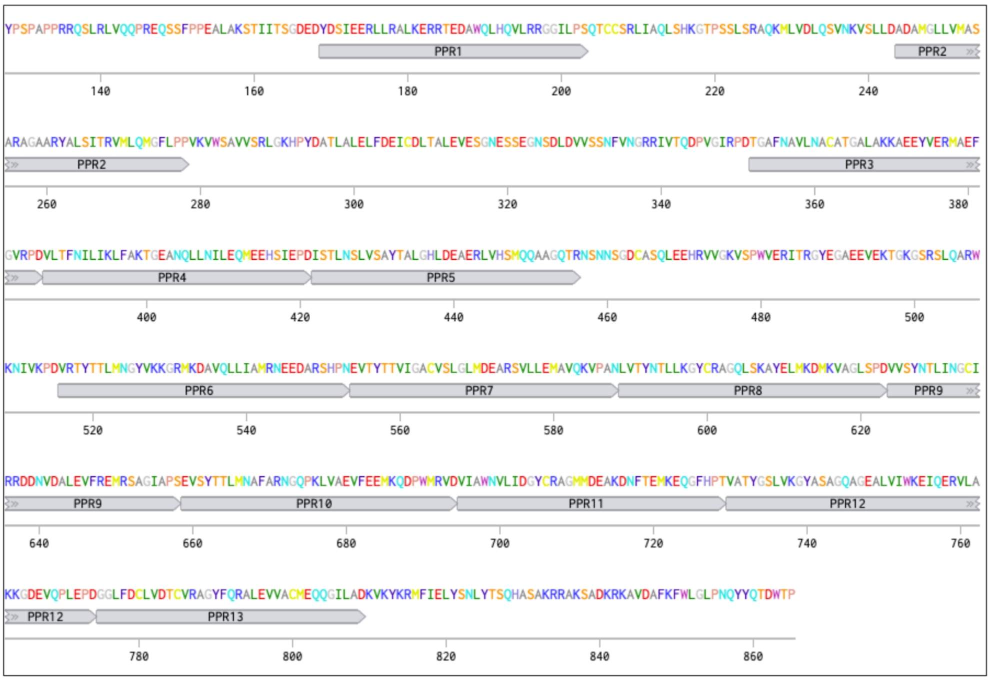

Mp1g13160.1

P	P	P	P	P	P	P	P	P	P	P	P	P	Type
E	G	N	N	N	T	T	N	N	T	N	G	D	5
S	P	D	D	R	N	N	D	S	D	T	D	D	Last
.	.	U	U	C	A	A	U	C	G	C	U	.	Match 5
		0.61	0.61	0.15	0.61	0.61	0.61	0.51	0.68	0.32	0.09		Prob
.	.	A	A	A,G,U	G	G	A	U	A,U	U	C	.	Match Last
		-0.61	-0.61	-0.05	-0.25	-0.25	-0.61	-0.10	-0.29	0.17	0.03		Prob

At HCF152

P	P	P	P	P	P	P	P	P	P	P	P	Type
G	N	N	H	T	T	N	N	T	N	G	D	5
H	D	C	C	D	N	D	T	D	N	D	N	Last
.	U	.	.	G	A	U	C	G	C	U	.	Match 5
	0.61			0.68	0.61	0.61	0.32	0.68	0.63	0.09		Prob
.	A	.	.	A,U	G	A	U	A,U	U	C	.	Match Last
	-0.61			-0.29	-0.25	-0.61	0.17	-0.29	0.16	0.03		Prob

b

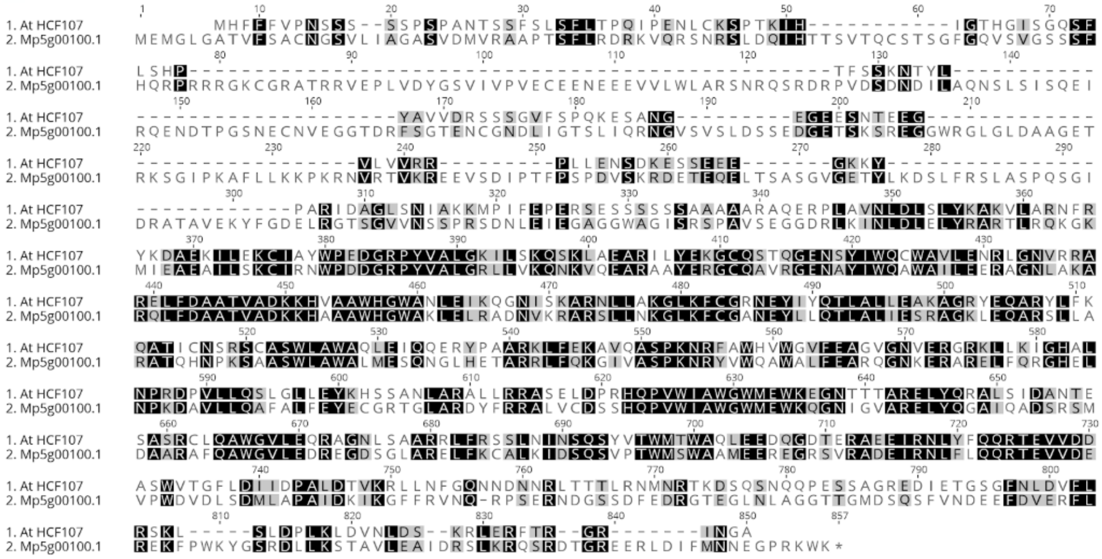

c

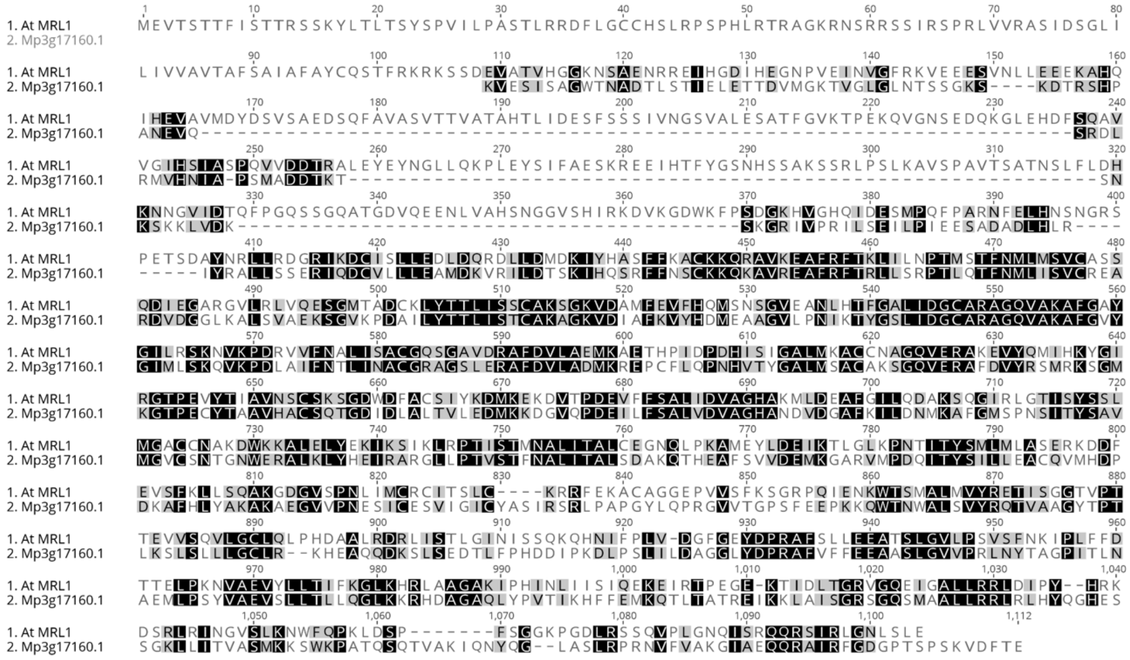

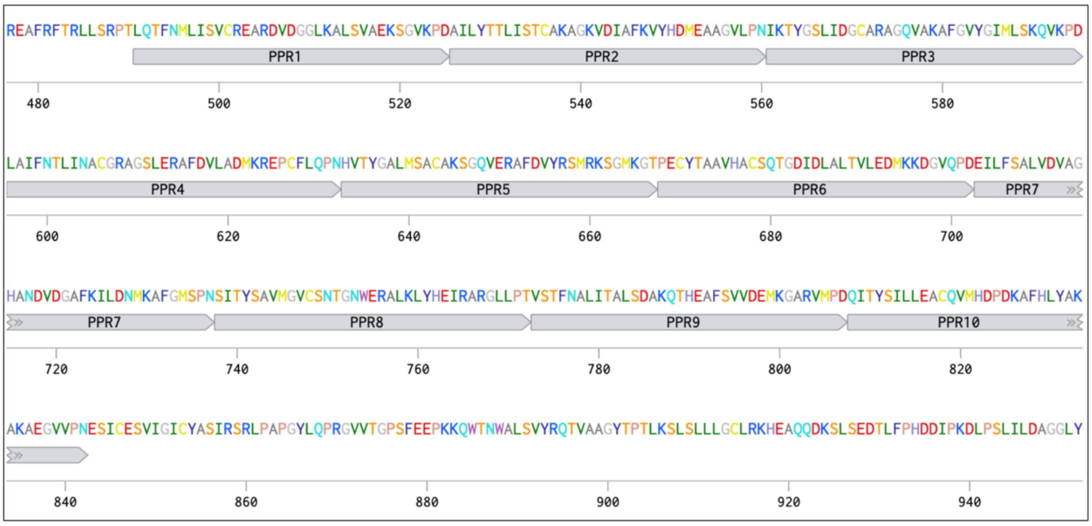

Mp3g17160.1

P	P	P	P	P	P	P	P	P	P	Type
N	T	G	N	G	T	S	S	N	S	5
D	N	D	N	T	D	N	T	D	N	Last
U	A	U	C	.	G	A	.	U	A	Match 5
0.61	0.61	0.09	0.63		0.68	0.44		0.61	0.44	Prob
A	G	C	U	.	A,U	C	.	A	C	Match Last
-0.61	-0.25	0.03	0.16		-0.29	-0.19		-0.61	-0.19	Prob

At MRL1

P	P	P	P	P	P	P	P	P	P	P	Type
S	N	T	G	N	G	T	S	S	N	S	5
M	D	N	D	D	T	D	G	T	N	N	Last
.	U	A	U	U	.	G	.	.	C	A	Match 5
	0.61	0.61	0.09	0.61		0.68			0.63	0.44	Prob
.	A	G	C	A	.	A,U	.	.	U	C	Match Last
	-0.61	-0.25	0.03	-0.61		-0.29			0.16	-0.19	Prob

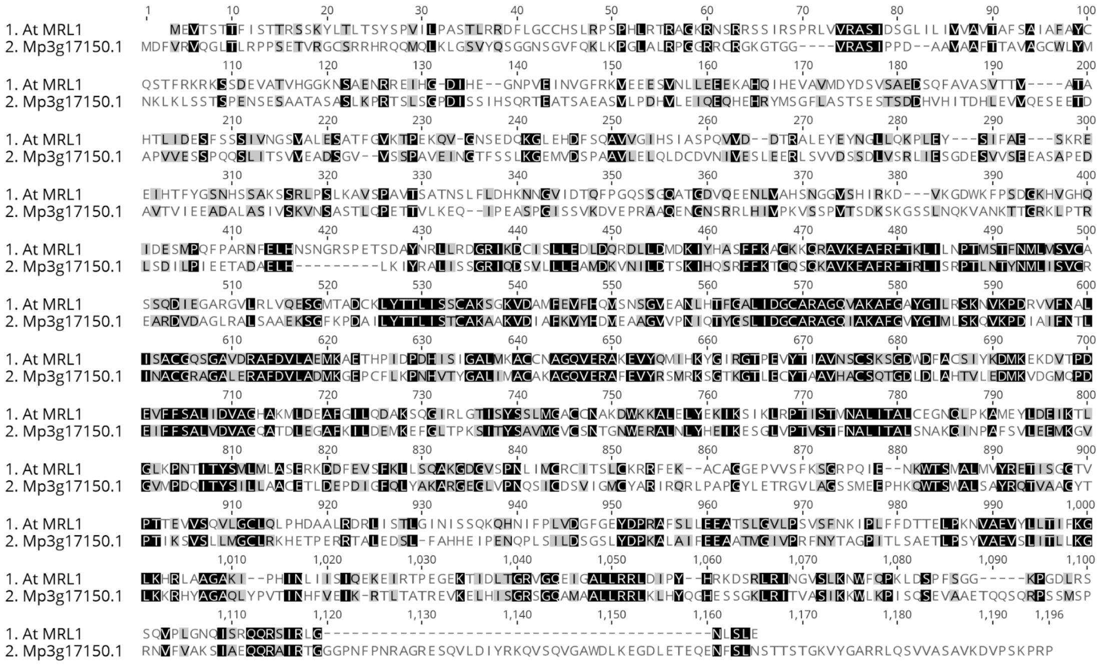

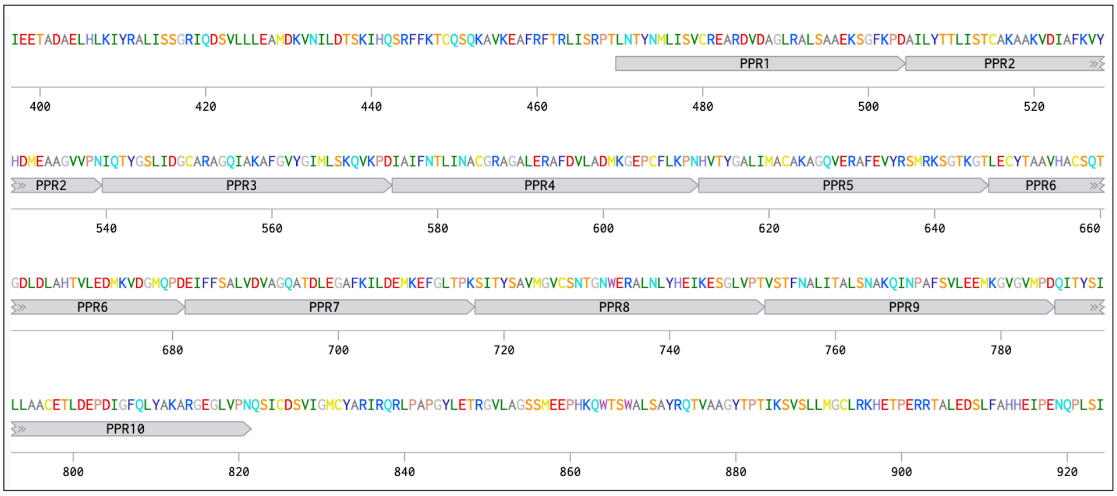

Mp3g17150.1

P	P	P	P	P	P	P	P	P	P	Type
N	T	G	N	G	T	S	S	N	S	5
D	N	D	N	T	D	K	T	D	N	Last
U	A	U	C	.	G	.	.	U	A	Match 5
0.61	0.61	0.09	0.63		0.68			0.61	0.44	Prob
A	G	C	U	.	A,U	.	.	A	C	Match Last
-0.61	-0.25	0.03	0.16		-0.29			-0.61	-0.19	Prob

At MRL1

P	P	P	P	P	P	P	P	P	P	P	Type
S	N	T	G	N	G	T	S	S	N	S	5
M	D	N	D	D	T	D	G	T	N	N	Last
.	U	A	U	U	.	G	.	.	C	A	Match 5
	0.61	0.61	0.09	0.61		0.68			0.63	0.44	Prob
.	A	G	C	A	.	A,U	.	.	U	C	Match Last
	-0.61	-0.25	0.03	-0.61		-0.29			0.16	-0.19	Prob

d

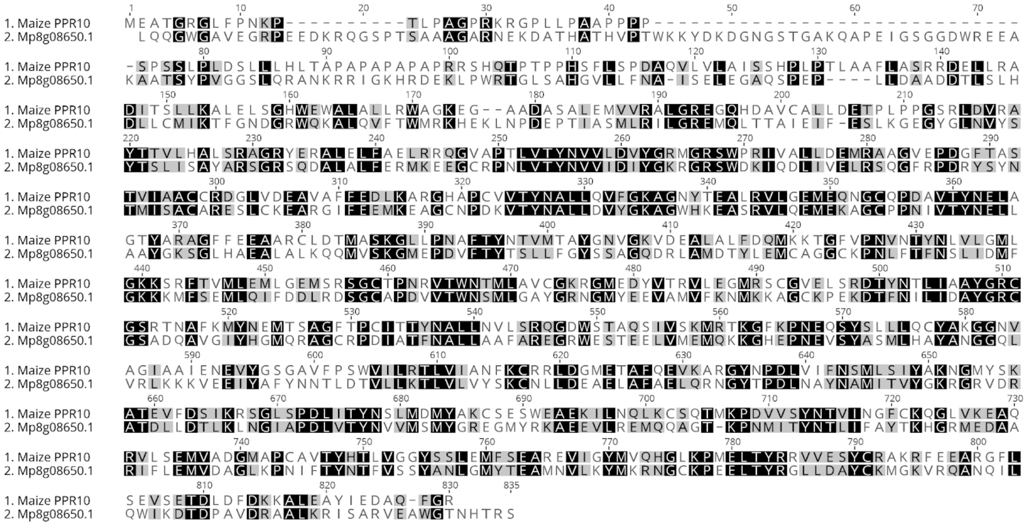

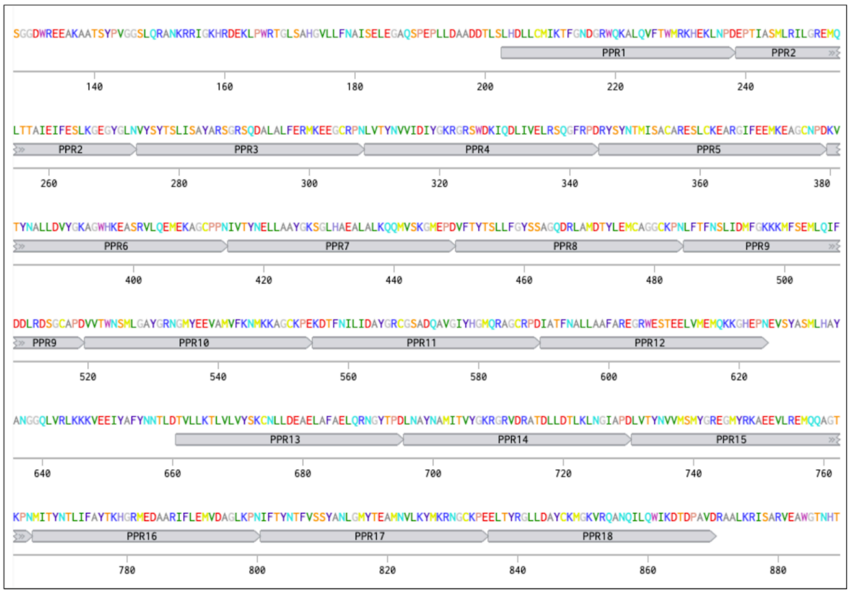

Mp8g08650.1

P	P	P	P	P	P	P	P	P	P	P	P	P	P	P	P	P	P	Type
L	A	T	N	N	N	N	T	N	N	N	N	K	N	N	N	N	R	5
D	N	N	D	D	N	D	N	D	E	D	N	D	D	N	N	E	D	Last
.	C	A	U	U	C	U	A	U	G	U	C	.	U	C	C	G	.	Match 5
	0.11	0.61	0.61	0.61	0.63	0.61	0.61	0.61	0.06	0.61	0.63		0.61	0.63	0.63	0.06		Prob
.	A	G	A	A	U	A	G	A	U	A	U	.	A	U	U	U	.	Match Last
	0.07	-0.25	-0.61	-0.61	0.16	-0.61	-0.25	-0.61	0.04	-0.61	0.16		-0.61	0.16	0.16	0.04		Prob

ZmPPR10

P	P	P	P	P	P	P	P	P	P	P	P	P	P	P	P	Type
T	E	T	N	N	N	N	N	N	N	R	N	N	N	H	R	5
D	D	T	D	N	N	N	S	C	N	D	D	D	C	M	D	Last
G	.	.	U	C	C	C	C	.	C	.	U	U	.	.	.	Match 5
0.68			0.61	0.63	0.63	0.63	0.51		0.63		0.61	0.61				Prob
A,U	.	.	A	U	U	U	U	.	U	.	A	A	.	.	.	Match Last
-0.29			-0.61	0.16	0.16	0.16	-0.10		0.16		-0.61	-0.61				Prob

Figure S3: *Marchantia* HCF152, HCF107, MRL1 and PPR10 putative homologs
Marchantia PPR homolog predictions were made using Orthofinder [4]
a) Top: Amino acid sequence alignments of AtHCF152 (AT3G09650) and Mp*PPR_16* (Mp1g13160.1), using MUSCLE [5]. *Marchantia* HCF152 putative homolog PPR domain (middle) and binding site (bottom) prediction/comparison for Mp1g13160.1 and AtHCF152 based on Cheng et al. [6].
b) Top: Amino acid sequence alignments of AtHCF107 (AT3G17040) and Mp5g00100.1, using MUSCLE.
c) Top: Amino acid sequence alignments of AtMRL1 (AT4G34830) and the two *Marchantia* putative homologs, Mp*PPR_28* (Mp3g17160.1) and Mp*PPR_29* (Mp3g17150.1), using MUSCLE. *Marchantia* MRL1 homolog PPR domain (middle) and binding site (bottom) prediction/comparison for Mp3g17160.1/ Mp3g17150.1 and AtMRL1 based on Cheng et al.
d) Top: Amino acid sequence alignments of ZmPPR10 (AQK81740) and Mp*PPR_41* (Mp8g08650.1), using MUSCLE. *Marchantia* PPR10 putative homolog PPR domain (middle) and binding site (bottom) prediction/comparison for Mp8g08650.1 and ZmPPR10 based on Cheng et al.

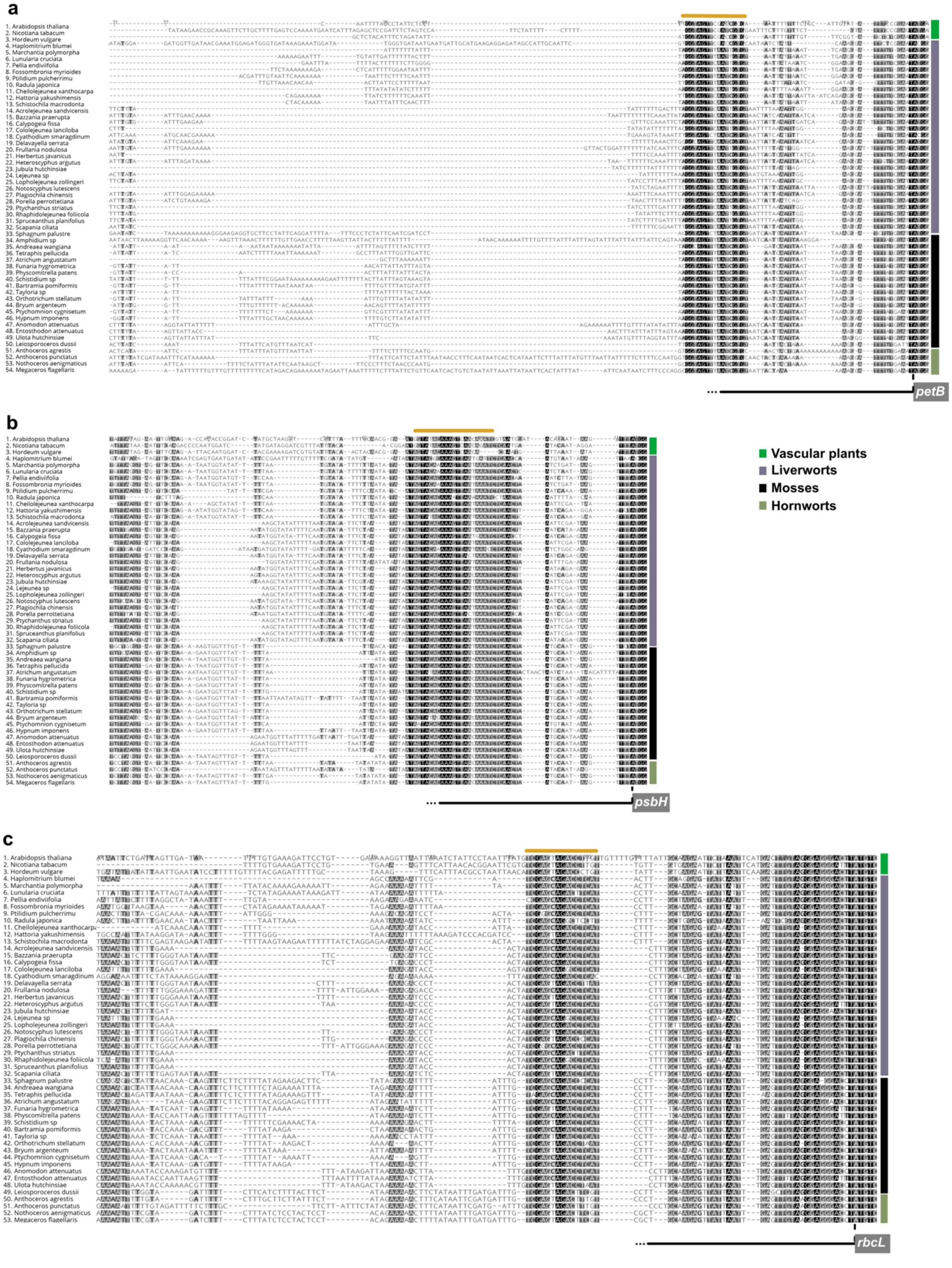
a
b
c
petB
psbH
rbcL
Vascular plants
Liverworts
Mosses
Hornworts

d

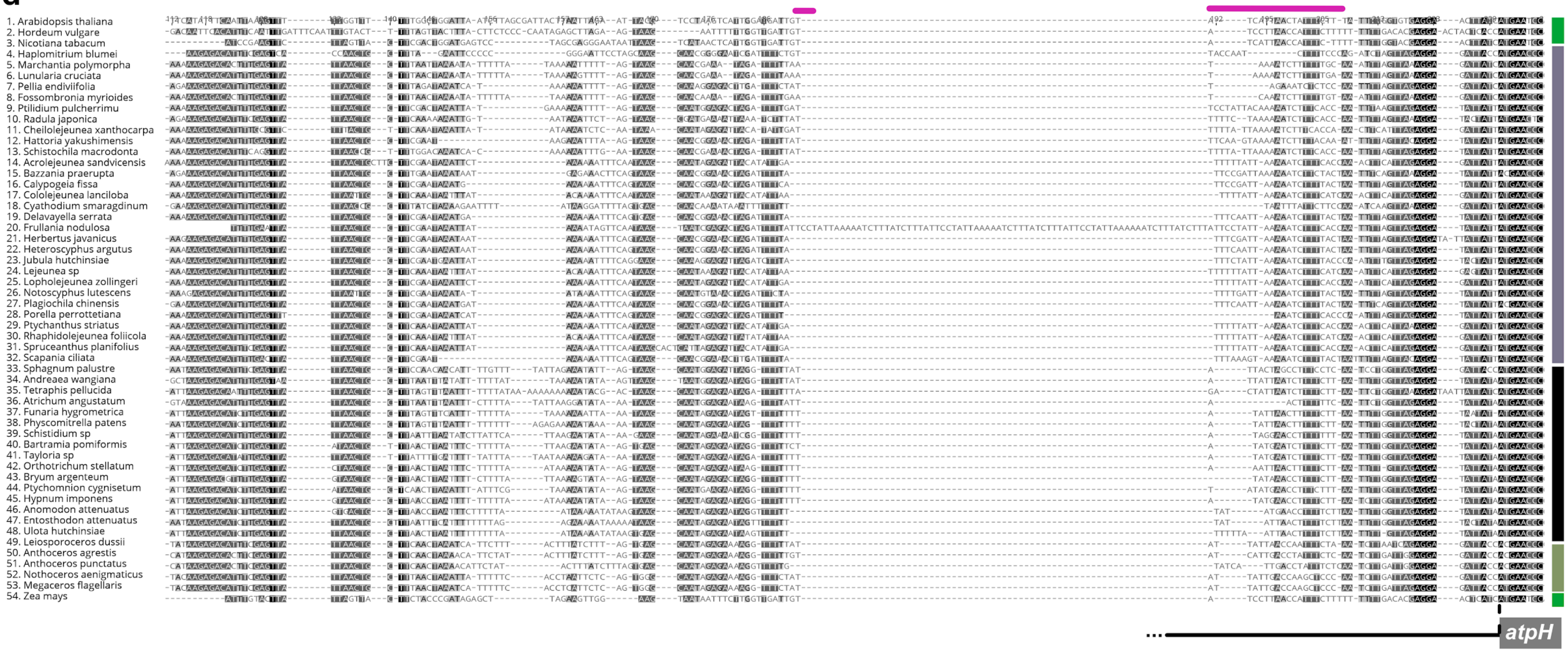
1. Arabidopsis thaliana
2. Hordeum vulgare
3. Nicotiana tabacum
4. Haplomitrium blumei
5. Marchantia polymorpha
6. Lunularia cruciata
7. Pellia endiviifolia
8. Fossombronia myrioides
9. Ptilidium pulcherrimu
10. Radula japonica
11. Cheilolejeunea xanthocarpa
12. Hattoria yakushimensis
13. Schistochila macrodonta
14. Acrolejeunea sandvicensis
15. Bazzania praerupta
16. Calypogeia fissa
17. Cololejeunea lanciloba
18. Cyathodium smaragdinum
19. Delavayella serrata
20. Frullania nodulosa
21. Herbertus javanicus
22. Heteroscyphus argutus
23. Jubula hutchinsiae
24. Lejeunea sp
25. Lopholejeunea zollingeri
26. Notoscyphus lutescens
27. Plagiochila chinensis
28. Porella perrottetiana
29. Ptychanthus striatus
30. Rhaphidolejeunea foliicola
31. Spruceanthus planifolius
32. Scapania ciliata
33. Sphagnum palustre
34. Andreaea wangiana
35. Tetraphis pellucida
36. Atrichum angustatum
37. Funaria hygrometrica
38. Physcomitrella patens
39. Schistidium sp
40. Bartramia pomiformis
41. Tayloria sp
42. Orthotrichum stellatum
43. Bryum argenteum
44. Ptychomnion cygnisetum
45. Hypnum imponens
46. Anomodon attenuatus
47. Entosthodon attenuatus
48. Ulota hutchinsiae
49. Leiosporoceros dussii
50. Anthoceros agrestis
51. Anthoceros punctatus
52. Nothoceros aenigmaticus
53. Megaceros flagellaris
54. Zea mays
atpH

Figure S4

Multiple sequence alignments of regions upstream the 5′ UTR of **(a)** *petB*, **(b)** *psbH*, **(c)** *rbcL* and **(d)** *atpH*, of the 51 bryophyte plastid genomes used in this study and key angiosperms, performed with MUSCLE [5]. ATG site is indicated with a dashed line. Coding sequence is indicated with a grey box. The predicted PPR binding site is highlighted by an orange line above alignments. Purple line above alignment in **(d)** indicates the PPR10 binding motif in angiosperms[7]. The colouring used for that column depends on the fraction of the column that is made of letters from this group. Black: 100% similar, dark-grey 80%-100% similar, lighter grey: 60%-80% similar, white: less than 60% similar. All letters from this group are assigned this one colour, and all letters outside of the group are not coloured.

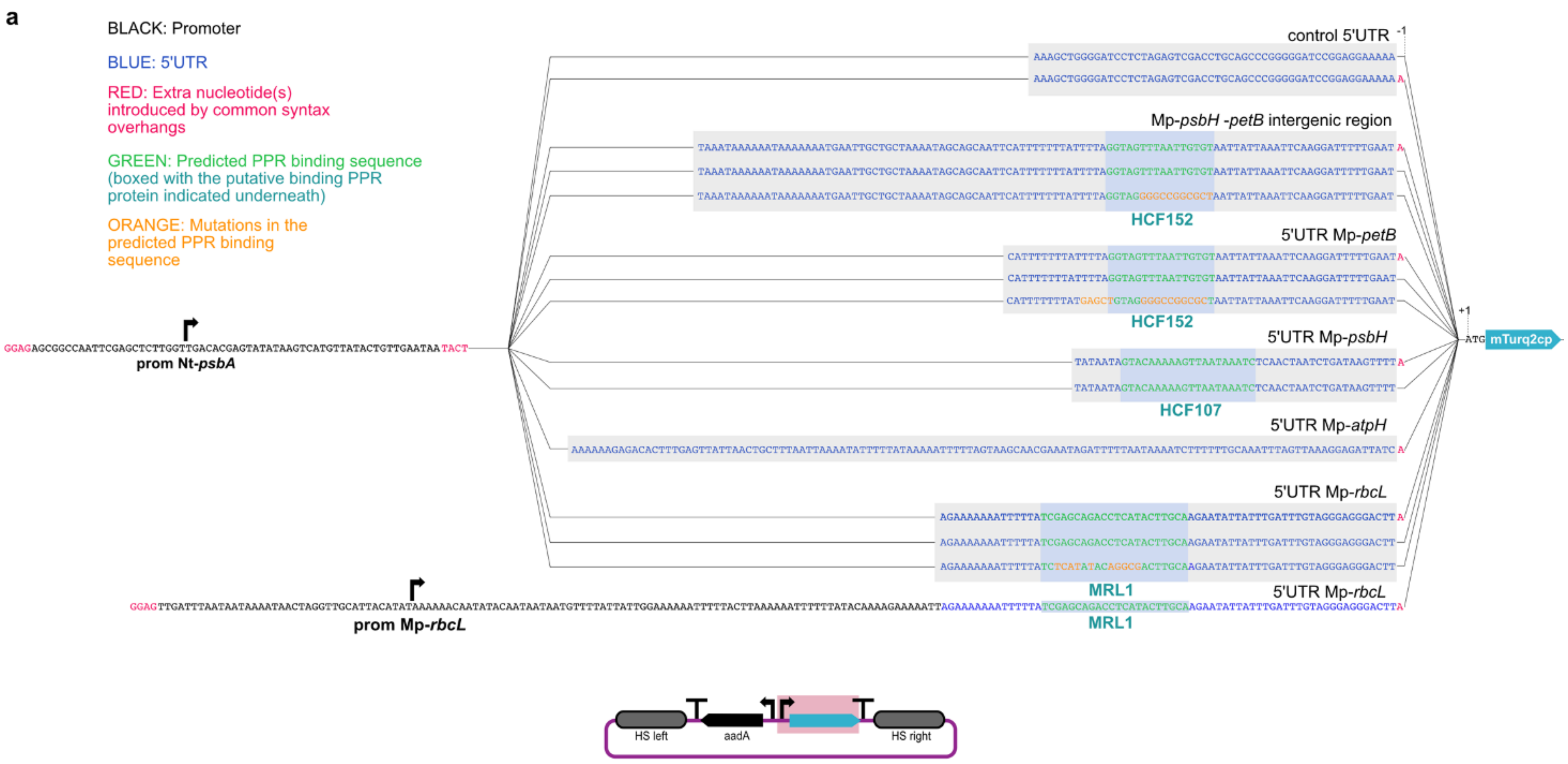
a
BLACK: Promoter
BLUE: 5'UTR
RED: Extra nucleotide(s) introduced by common syntax overhangs
GREEN: Predicted PPR binding sequence (boxed with the putative binding PPR protein indicated underneath)
ORANGE: Mutations in the predicted PPR binding sequence
prom Nt-psbA
prom Mp-rbcL
control 5'UTR
-1
Mp-psbH -petB intergenic region
HCF152
5'UTR Mp-petB
HCF152
5'UTR Mp-psbH
HCF107
5'UTR Mp-atpH
5'UTR Mp-rbcL
MRL1
5'UTR Mp-rbcL
MRL1
+1
ATG
mTurq2cp
HS left
aadA
HS right

b

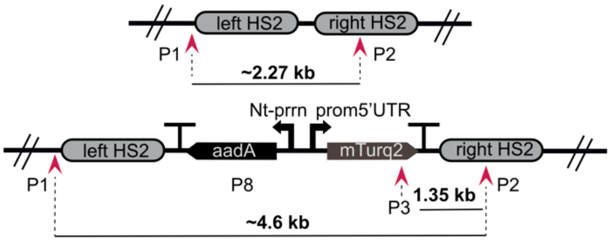
left HS2
right HS2
P1
P2
~2.27 kb
Nt-prrn
prom5'UTR
aadA
mTurq2
P8
P3
1.35 kb
~4.6 kb

c

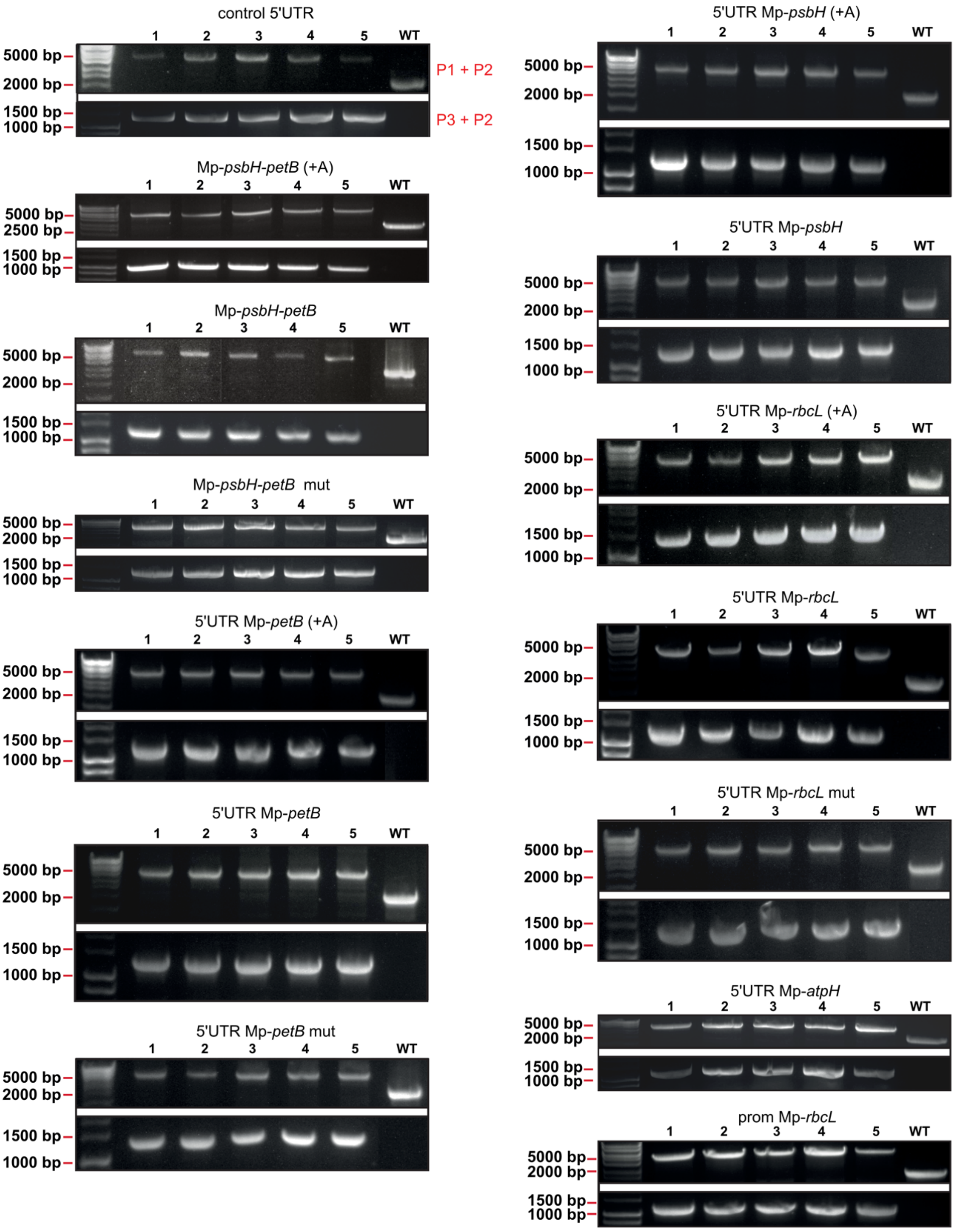
control 5'UTR
1 2 3 4 5 WT
5000 bp
2000 bp
1500 bp
1000 bp
P1 + P2
P3 + P2
Mp-psbH-petB (+A)
2500 bp
Mp-psbH-petB
Mp-psbH-petB mut
5'UTR Mp-petB (+A)
5'UTR Mp-petB
5'UTR Mp-petB mut
5'UTR Mp-psbH (+A)
5'UTR Mp-psbH
5'UTR Mp-rbcL (+A)
5'UTR Mp-rbcL
5'UTR Mp-rbcL mut
5'UTR Mp-atpH
prom Mp-rbcL

Figure S5

a) Schematic representation of different constructs

Bottom: Schematic representation of a L2 Loop construct to express the chloroplast codon optimized mTurq2cp fluorescent protein under the control of the tobacco Nt-*psbA* promoter and different combinations of PPR binding sequences (top figure – pink box in the plasmid map) using the left and right homologous sequences for integration in the chloroplast *rbcL−trnR* intergenic region. Control 5'UTR (+A) construct from [7].

b-c) Validation of homoplasmy

b) Schematic representation of the *rbcL-trnR* target region (flanked by left homologous sequence 2 (HS2) and right homologous sequence 2 (HS2) [8]) in the wild type chloroplast genome (top) and the same region after integration of the DNA construct (bottom). Red arrowheads indicate the position of the PCR primers used for the detection of wild type or homoplastic transplastomic lines. Maps not to scale. **c)** PCR analysis of genomic DNA isolated from wild type and transplastomic plants. Homoplasmy (bottom, primers P1+P2) and integrity of the reporter gene (top, primers P2+P3) were confirmed for transplastomic lines after 2 months of subculture under selective conditions. The primer pair used for each PCR are shown next to the gel images.

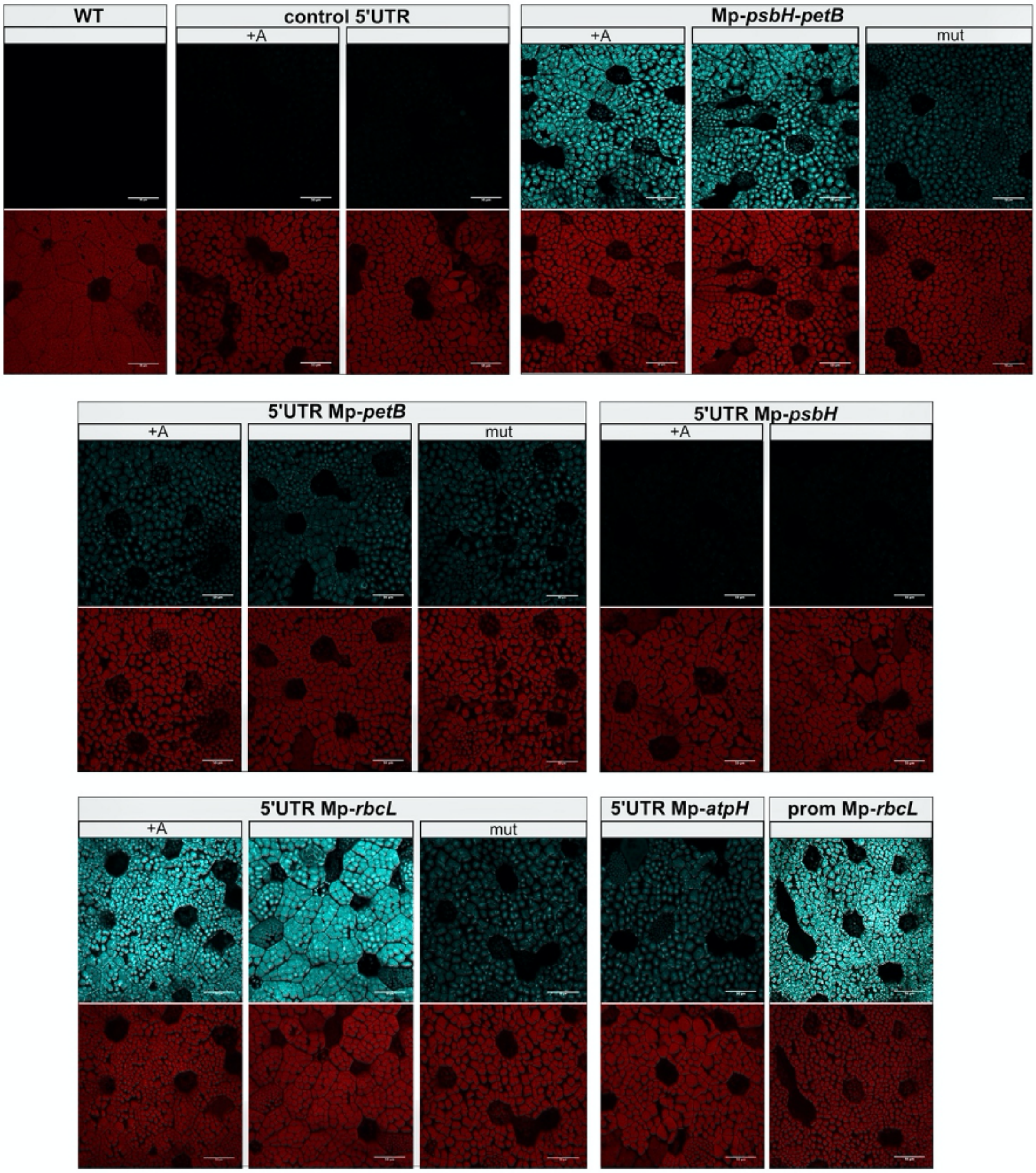
WT
control 5'UTR
+A
Mp-psbH-petB
+A
mut
5'UTR Mp-petB
+A
mut
5'UTR Mp-psbH
+A
5'UTR Mp-rbcL
+A
mut
5'UTR Mp-atpH
prom Mp-rbcL

Figure S6 Confocal microscopy images of *Marchantia* transplastomic 3-day gemmae expressing the mTurq2cp fluorescent protein under the control of the Nt-*psbA* promoter fused to different candidate stabilisation sequences

Control 5'UTR, Mp-*psbH-petB,* 5'UTR Mp-*petB*, 5'UTR Mp-*psbH,* 5'UTR Mp-*rbcL*, 5'UTR Mp-*atpH* and the promoter Mp-*rbcL*. +A: Adenine introduced by the common syntax present between the 5'UTR and the mTurq2cp coding sequence. Mut: predicted PPR binding sequence mutated. Panel top: Chlorophyll autofluorescence channel, Panel bottom: mTurq2cp channel. All images acquired using identical instrument settings. 5'UTR Mp-*rbcL* and prom Mp-*rbcL* confers the highest levels of expression followed by Mp-*psbH-petB*, 5'UTR Mp-*petB* and 5'UTR Mp-*atpH.* 5'UTR Mp-*psbH* expression levels are similar to those of the control 5'UTR. The addition of an extra "A" between the 5'UTR and the mTurq2cp coding sequence does not significantly affect the expression of mTurq2cp. Expression levels are reduced when the predicted PPR binding sequences are mutated. Control 5'UTR (+A) construct lines from [7]

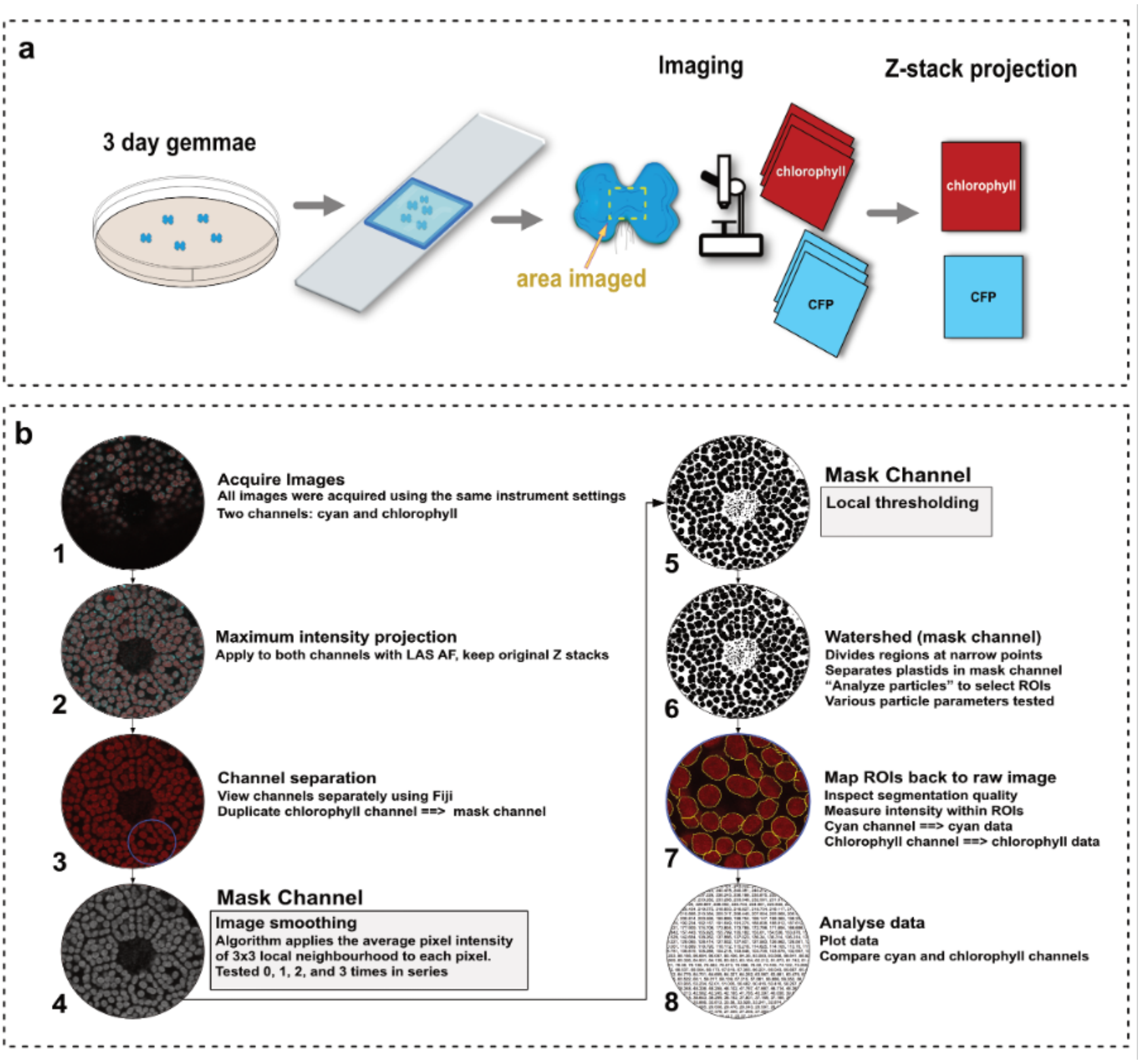

c

Macro (plain text between the bold start and stop lines)

```
run("Duplicate...", " ");
run("Smooth");
run("Smooth");
run("Auto Local Threshold", "method=Phansalkar radius=15 parameter_1=0 parameter_2=0 white");
run("Watershed");
run("Clear Results");
run("Analyze Particles...", "size=250-1500 circularity=0.60-1.00 display exclude clear add");
close();
run("Clear Results");
roiManager("Measure");
String.copyResults();
run("Clear Results");
\\ end
```

Figure S7: **Schematic of sample preparation and plastid segmentation pipeline**
a) Gemmae were plated on half strength Gamborg B5 1.2% (w/v) agar plates and placed in a growth cabinet for 3 days under continuous light at 21 ºC. A gene frame was positioned on a glass slide and 20 µL of half strength Gamborg B5 1.2% (w/v) agar were placed within the gene frame. 5 gemmae were then placed within the media filled gene frame, 20 µL of milliQ water was added and then a cover slip was used to seal the geneframe. Plants were then imaged immediately using an SP8 fluorescent confocal microscope.

b) Image processing pipeline. Steps 1 and 2 were performed on the Leica SP8 microscope, steps 3-7 were performed in Fiji using a custom macro (included in supplement), step 8 was performed using Google Sheets for spreadsheet data-preparation and R Studio for statistical analysis. All images were acquired using identical instrument settings. Images consisted of 16 Z stacks of 3 µm thickness.

c) Fiji Macro.

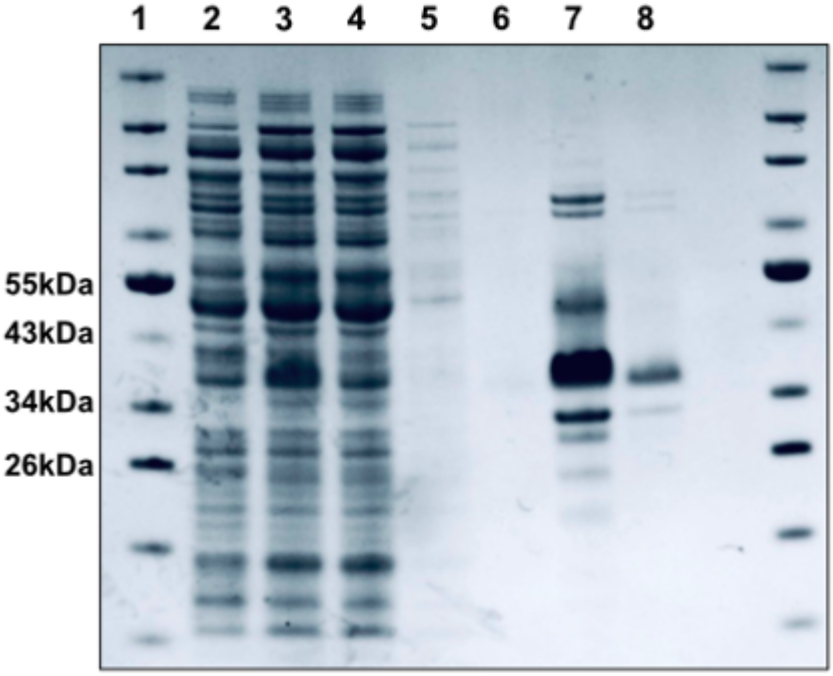

Figure S8
Coomassie Blue–Stained Protein Gel of mTurquoise2 recombinant protein and its purification
Lane 1 PageRuler Pre-stained NIP protein ladder (#26635, ThermoFisher); lane 2 total cell extract from non transformed cells; lane 3 total extract from IPTG-induced bacteria cells; lane 4 flow-through in the first step of the protein purification; lane 5 and 6, washes of 1 and 2 respectively. Lane 7, purified mTurquoise2 recombinant protein in the first and second (lane 8) elution fraction.

Sample	Total Reads	Forward mapped reads	Reverse mapped reads
A-TEX	10.636.145	6,310,844	3,566,280
A	9.988.769	6,208,786	5,291,442
B-TEX	8.517.797	5,109,165	1,883,046
B	9.063.993	2,303,814	4,747,933

Table S1 Differential RNA-sequencing mapping statistics

Table S2: **List of TSSs identified using dRNAseq**
Separate excel file

Table S3: **List of MEME identified promoter motifs**
Separate excel file

	Liverworts	Order	Family	Genbank Accession
1	*Cheilolejeunea xanthocarpa*	Porellales	Lejeuneaceae	MH064504
2	*Cololejeunea lanciloba*	Porellales	Lejeuneaceae	MH064505
3	*Schistochila macrodonta*	Jungermanniales	Schistochilaceae	MH064506
4	*Porella perrottetiana*	Porellales	Porellaceae	MH064507
5	*Radula japonica*	Porellales	Radulaceae	MH064508
6	*Jubula hutchinsiae*	Porellales	Jubulaceae	MH064509
7	*Frullania nodulosa*	Porellales	Frullaniaceae	MH064510
8	*Plagiochila chinensis*	Jungermanniales	Plagiochilaceae	MH064511
9	*Bazzania praerupta*	Jungermanniales	Lepidoziaceae	MH064512
10	*Scapania ciliata*	Jungermanniales	Scapaniaceae	MH064513
11	*Calypogeia fissa*	Jungermanniales	Calypogeiaceae	MH064514
12	*Heteroscyphus argutus*	Jungermanniales	Lophocoleaceae	MH064515
13	*Lunularia cruciata*	Marchantiales	Lunulariaceae	MW429511
14	*Lejeunea sp*	Porellales	Lejeuneaceae	MW429495
15	*Spruceanthus planifolius*	Porellales	Lejeuneaceae	MW429496
16	*Acrolejeunea sandvicensis*	Porellales	Lejeuneaceae	MW429497
17	*Rhaphidolejeunea foliicola*	Porellales	Lejeuneaceae	MW429498
18	*Ptychanthus striatus*	Porellales	Lejeuneaceae	MW429500
19	*Lopholejeunea zollingeri*	Porellales	Lejeuneaceae	MW429501
20	*Herbertus javanicus*	Jungermanniales	Herbertaceae	MW429507
21	*Delavayella serrata*	Jungermanniales	Delavayellaceae	MW429508

22	*Cyathodium smaragdinum*	Marchantiales	Cyathodiaceae	MW429509
23	*Fossombronia myrioides*	Fossombroniales	Fossombroniaceae	MW429510
24	*Hattoria yakushimensis*	Jungermanniales	Scapaniaceae	MW429512
25	*Notoscyphus lutescens*	Jungermanniales	Notoscyphaceae	MW429513
26	*Haplomitrium blumei*	Haplomitriales	Haplomitriaceae	MH064516
27	*Pellia endiviifolia*	Pelliales	Pelliaceae	NC_019628.1
28	*Ptilidium pulcherrimum*	Ptilidiales	Ptilidiaceae	HM222519.1
29	*Marchantia polymorpha*	Marchantiales	Marchantiaceae	MH635409.1
	**Mosses**	**Order**	**Family**	**Genbank Accession**
30	*Bryum argenteum*	Bryales	Bryaceae	MW602653
31	*Sphagnum palustre*	Sphagnales	Sphagnaceae	MW822172
32	*Atrichum angustatum*	Polytrichales	Polytrichaceae	MW556444
33	*Tetraphis pellucida*	Tetraphidales	Tetraphidaceae	MW822173
34	*Funaria hygrometrica*	Funariales	Funariaceae	MW648546
35	*Entosthodon attenuatus*	Funariales	Funariaceae	MW646101
36	*Bartramia pomiformis*	Bartramiales	Bartramiaceae	MW575014
37	*Ulota hutchinsiae*	Orthotrichales	Orthotrichaceae	MW822174
38	*Orthotrichum stellatum*	Orthotrichales	Orthotrichaceae	MW822170
39	*Ptychomnion cygnisetum*	Ptychomniales	Ptychomniaceae	MW822171
40	*Hypnum imponens (Callicladium imponens)*	Hypnales	Callicladiaceae	MW822169

41	*Anomodon attenuatus*	Hypnales	Anomodontaceae	MW528223
42	*Andreaea wangiana*	Andreaeales	Andreaeaceae	MW429499
43	*Amphidium sp*	Orthotrichales	Orthotrichaceae	MW429503
44	*Schistidium sp*	Grimmiales	Grimmiaceae	MW429504
45	*Tayloria sp*	Splachnales	Splachnaceae	MW429505
46	*Physcomitrella patens*	Funariales	Funariaceae	NC_005087.2
	**Hornworts**	**Order**	**Family**	**Genbank Accession**
47	*Megaceros flagellaris*	Dendrocerotales	Dendrocerotaceae	MW429502
48	*Anthoceros agrestis*	Anthocerotales	Anthocerotaceae	NC_049002.1
49	*Anthoceros punctatus*	Anthocerotales	Anthocerotaceae	NC_049001.1
50	*Leiosporoceros dussii*	Leiosporocerotales	Leiosporocerotaceae	NC_039750.1
51	*Nothoceros aenigmaticus*	Dendrocerotales	Dendrocerotaceae	NC_020259.1

Table S4: Bryophyte plastid genomes used in this study

Name	assembly	length bp
Anomodon attenuatus	complete	115682
Atrichum angustatum	complete	116350
Bartramia pomiformis	complete	116167
Bryum argenteum	complete (small repeats)	114181
Entosthodon attenuatus	complete (one repeat)	113759
Funaria hygrometrica	complete (one repeat)	113244
Hypnum imponens	complete	115808
Orthotrichum stellatum	complete	113708
Ptychomnion cygnisetum	complete	114393
Sphagnum palustre	complete	128848
Tetraphis pellucida	complete	117992
Ulota hutchinsiae	complete (not circular)	114069

Table S5: Moss genome assemblies overview

Table S6: List of constructs *Separate excel file*

Table S7: Microscopy image quantification *Separate excel file*

Description - For amplifcation of:	Part type	Fw primer	5'->3'	Rev primer	5'->3'	Template
**Mp cam1 rbcL promoter region**		EF240	CCAACACCTGCTTTAGTCTCCG	EF239	GGAAAAATTGTTAAGGTCAATGTGTATC	gDNA
**Mp cam1 rbcL promoter region domesticated**	PROM5	EF616	TAGAGCTCTTCGTCTCTGGAGTTGATTTAATAATAAAATAACTAGG	EF269	ACTAGCTCTTCGTCTCACATTAAGTCCCTCCCTACAAATC	Mp cam1 rbcL promoter region
**Mp cam1 atpH promoter region**		EF223	AGGCGAATCTATGGAAGGGCAT	EF224	CTTCTGCTTCAGGCTGTCTTGC	gDNA
**5'UTR Mp-atpH**	5UTR	AT001	taGAGCTCTTCgtctcTTACTAAAAAAGAGACACTTTGAGTTATTAACTGC	EF610	AGCAGCTCTTCGTCTCTCCATGATAATCTCCTTTAACTAAATTTGCAAAAAAG	Mp cam1 atpH promoter region
**Mp-psbH-petB**	5UTR	AT012	taGAGCTCTTCgtctcTTACTTAAATAAAAAATAAAAAAATGAATTGCTGCTAAAATAGC	AT011	AGCAGCTCTTCGTCTCTCATTATTCAAAAATCCTTGAATTTAATAATTACACAATTAAAC	gDNA
**Mp-psbH-petB (-A)**	5UTR-A	AT012	taGAGCTCTTCgtctcTTACTTAAATAAAAAATAAAAAAATGAATTGCTGCTAAAATAGC	EF612	AGCAGCTCTTCGTCTCTcCATATTCAAAAATCCTTGAATTTAATAATTACAC	Mp-psbH-petB
**Mp-psbH-petB**	5UTR	AT012	taGAGCTCTTCgtctcTTACTTAAATAAAAAATAAAAAAATGAATTGCTGCTAAAATAGC	EF615	AGCAGCTCTTCGTCTCTCCATATTCAAAAATCCTTGAATTTAATAATTAGCGCCGGCCCCTACCTAAAA	Mp-psbH-petB
**5'UTR Mp-petB**	5UTR	PP01	taGAGCTCTTCgtctcTTACTCATTTTTTTATTTTAGGTAGTTTAATTGTG	PP02	AGCAGCTCTTCGTCTCTCATTATTCAAAAATCCTTGAATTTAATAATTAC	gDNA
**5'UTR Mp-petB**	5UTR-A	PP01	taGAGCTCTTCgtctcTTACTCATTTTTTTATTTTAGGTAGTTTAATTGTG	PP03	AGCAGCTCTTCGTCTCTcCATATTCAAAAATCCTTGAATTTAATAATTAC	5'UTR Mp-petB
**5'UTR Mp-petB mutated**	5UTR-A	PP04	taGAGCTCTTCgtctcTTACTCATTTTTTTATGAGCTGTAG	PP05	AGCAGCTCTTCGTCTCTcCATATTCAAAAATCCTTGAATTTAATAATTAGCGCCGGCCC	5'UTR Mp-petB
**5'UTR Mp-psbH**	5UTR	PP06	taGAGCTCTTCgtctcTTACTTATAATAGTACAAAAAGTTAATAAATC	PP07	AGCAGCTCTTCGTCTCTCATTAAAACTTATCAGATTAGTTG	gDNA
**5'UTR Mp-psbH**	5UTR-A	PP06	taGAGCTCTTCgtctcTTACTTATAATAGTACAAAAAGTTAATAAATC	PP08	AGCAGCTCTTCGTCTCTcCATAAAACTTATCAGATTAGTTG	5'UTR Mp-psbH
**5'UTR Mp-rbcL**	5UTR	MRL1-F	TAGAGCTCTTCGTCTCTTACTAGAAAAAAAATTTTTATCGAGCAGACCTC	MRL1-R1	AGCAGCTCTTCGTCTCTCATTAAGTCCCTCCCTACAAATCAAATAATATTCTTG	gDNA
**5'UTR Mp-rbcL**	5UTR-A	MRL1-F	TAGAGCTCTTCGTCTCTTACTAGAAAAAAAATTTTTATCGAGCAGACCTC	MRL1-R2	AGCAGCTCTTCGTCTCTCCATAAGTCCCTCCCTACAAATCAAATAATATTCTTG	5'UTR Mp-rbcL
**5'UTR Mp-rbcL mutated**	5UTR-A-SCR	MRL1-scr-F3	TAGAGCTCTTCGTCTCTTACTAGAAAAAAAATTTTTATCTCATATACAGGCGACTTG	MRL1-R3	AGCAGCTCTTCGTCTCTCCATAAGTCCCTCCCTACAAATCAAATAATATTC	5'UTR Mp-rbcL
**Nt-psbA (-A)**	PROM5-A	AT004	taGAGCTCTTCgtctcTGGAGagcggccaattcgagctc	EF614	AGCAGCTCTTCGTCTCTcCATTTTTTCCTCCGGATCCCCCGG	pNt-psbA-control 5'UTR:mTurq2cp
**mTurq2cp (-A)**	CDS-A	EF613	taGAGCTCTTCgtctcTatggtttctaaaggagaagaacttttttactg	EF611	CAGCTCTTCGTCTCTAAGCttatttgtacaattcatccattcctaatg	pNt-psbA-control 5'UTR:mTurq2cp
**Nt-psbA (-A)**	PROM	AT004	TAGAGCTCTTCGTCTCTGGAGAGCGGCCAATTCGAGCTC	AT005c	AGCAGCTCTTCGTCTCTAGTATTATTCAACAGTATAACATGACTTATATACTCG	pNt-psbA-control 5'UTR:mTurq2cp

**Genotyping primers**						
		KM14 (P1)	CGGAGACTAAAGCAGGTGTTGG	EF513 (P2)	ACAGATCCCATACTACCGCC	
**P1, P2 and P3 in Supp Fig. 5**						
		EF344 (P3)	ttacctgtaccatggcctac			

Table S8: List of primers used in this study

REFERENCES

(1) Lohse, M., Drechsel, O., Kahlau, S., and Bock, R. (2013) Organellar Genome DRAW—a suite of tools for generating physical maps of plastid and mitochondrial genomes and visualizing expression data sets. *Nucleic Acids Res. 41*, 575–581.

(2) Bowman, J. L., Kohchi, T., Yamato, K. T., Jenkins, J., Shu, S., Ishizaki, K., Yamaoka, S., Nishihama, R., Nakamura, Y., Berger, F., et al. (2017) Insights into Land Plant Evolution Garnered from the Marchantia polymorpha Genome. *Cell 171*, 287–304.e15.

(3) Darling, A. C. E., Mau, B., Blattner, F. R., and Perna, N. T. (2004) Mauve: multiple alignment of conserved genomic sequence with rearrangements. *Genome Res. 14*, 1394–1403.

(4) Emms, D. M., and Kelly, S. (2015) OrthoFinder: solving fundamental biases in whole genome comparisons dramatically improves orthogroup inference accuracy. *Genome Biol. 16*, 157.

(5) Edgar, R. C. (2004) MUSCLE: a multiple sequence alignment method with reduced time and space complexity. *BMC Bioinformatics 5*, 113.

(6) Cheng, S., Gutmann, B., Zhong, X., Ye, Y., Fisher, M. F., Bai, F., Castleden, I., Song, Y., Song, B., Huang, J., et al (2016) Redefining the structural motifs that determine RNA binding and RNA editing by pentatricopeptide repeat proteins in land plants. *Plant J. 85*, 532–547.

(7) Zhelyazkova, P., Hammani, K., Rojas, M., Voelker, R., Vargas-Suárez, M., Börner, T., and Barkan, A. (2012) Protein-mediated protection as the predominant mechanism for defining processed mRNA termini in land plant chloroplasts. *Nucleic Acids Res. 40*, 3092–3105.

(8) Sauret-Güeto, S., Frangedakis, E., Silvestri, L., Rebmann, M., Tomaselli, M., Markel, K., Delmans, M., West, A., Patron, N. J., and Haseloff, J. (2020) Systematic Tools for Reprogramming Plant Gene Expression in a Simple Model. *ACS Synth. Biol. 9*, 864–882.
